# Sequential Analysis of *Trans*-SNARE Formation in Intracellular Membrane Fusion

**DOI:** 10.1371/journal.pbio.1001243

**Published:** 2012-01-17

**Authors:** Kannan Alpadi, Aditya Kulkarni, Veronique Comte, Monique Reinhardt, Andrea Schmidt, Sarita Namjoshi, Andreas Mayer, Christopher Peters

**Affiliations:** 1 Verna and Marrs McLean Department of Biochemistry and Molecular Biology, Baylor College of Medicine, Houston, Texas, United States of America; 2 Département de Biochimie, Université de Lausanne, Epalinges, Switzerland; Princeton University, United States of America

## Abstract

SNARE complexes are required for membrane fusion in the endomembrane system. They contain coiled-coil bundles of four helices, three (Q_a_, Q_b_, and Q_c_) from target (t)-SNAREs and one (R) from the vesicular (v)-SNARE. NSF/Sec18 disrupts these *cis*-SNARE complexes, allowing reassembly of their subunits into *trans*-SNARE complexes and subsequent fusion. Studying these reactions in native yeast vacuoles, we found that NSF/Sec18 activates the vacuolar *cis*-SNARE complex by selectively displacing the vacuolar Q_a_ SNARE, leaving behind a Q_bc_R subcomplex. This subcomplex serves as an acceptor for a Q_a_ SNARE from the opposite membrane, leading to Q_a_-Q_bc_R trans-complexes. Activity tests of vacuoles with diagnostic distributions of inactivating mutations over the two fusion partners confirm that this distribution accounts for a major share of the fusion activity. The persistence of the Q_bc_R *cis*-complex and the formation of the Q_a_-Q_bc_R trans-complex are both sensitive to the Rab-GTPase inhibitor, GDI, and to mutations in the vacuolar tether complex, HOPS (HOmotypic fusion and vacuolar Protein Sorting complex). This suggests that the vacuolar Rab-GTPase, Ypt7, and HOPS restrict *cis*-SNARE disassembly and thereby bias *trans*-SNARE assembly into a preferred topology.

## Abstract 2

SM proteins stabilize *cis*-SNARE complexes leading to a specific preferred topology for *trans*-SNARE formation.

## Author Summary

Cellular components often travel between organelles in vesicular entities. This intracellular traffic usually involves production of a vesicle containing cargo from one organelle membrane, movement of the vesicle to its destination, and then fusion of the vesicle with the target organelle. Thus, membrane fusion is a fundamental process required for these intracellular trafficking events. SNARE proteins and SM proteins mediate this fusion process. SNAREs form complexes that are either located on the same membrane or vesicle (called *cis*-SNARE complexes) or bridge two membrane compartments or vesicles (*trans*-SNARE complexes). The *cis*-SNARE complexes must be activated before *trans*-SNARE complexes can form and allow the membranes to fuse. We investigated the mechanism of *cis*-SNARE activation and *trans*-SNARE formation by studying the fusion of highly purified yeast vacuoles. We found that *cis*-SNARE activation involves the selective removal of a single SNARE protein from a pre-existing *cis*-SNARE complex, which is replaced by a similar SNARE originating from the other fusion partner. The activated *cis*-SNARE complexes depended on SM proteins for their stability. Thus, we have shown that the preferred topology of *trans*-SNARE formation is determined by *cis*-SNARE–SM protein interactions.

## Introduction

Cognate combinations of v- and t-SNAREs mediate membrane docking in every vesicular transport step in the endomembrane system. v- and t-SNAREs bind each other in coiled-coil complexes containing four helices [1]. Three of these helices (termed Q_a_, Q_b_, and Q_c_) are provided by t-SNAREs, while one (termed R) is provided by the v-SNARE [2],[3]. SNAREs from the two fusing membranes form trans-complexes between the membranes [4],[5]. It is clear that these trans-complexes are necessary for subsequent fusion; however, the pathway leading to their establishment is less well understood. Important observations have been obtained from experiments with purified SNAREs, studied either in detergent solution or after reconstitution into artificial lipid bilayers [4]. SNAREs form four-helix Q_abc_R complexes. They are a substrate for the ATP-driven chaperone Sec18/NSF, which completely disrupts pure SNARE complexes [6],[7]. Singular SNAREs can re-associate into complexes. Depending upon the method chosen, this re-association can occur in different topologies.

In most in vitro studies with reconstituted SNAREs in proteoliposomes, fusion-active combinations of SNAREs were found to require the Q_a_, Q_b_, and Q_c_-SNARE in one membrane and the R-SNARE on the other vesicle [4],[8]–[10]. The preference for this contribution is consistent with the fact that Q_abc_-containing SNARE complexes can form without an R-SNARE [3]. Thus, co-reconstituting these three Q-SNAREs into one liposome likely favors the formation of this Q_abc_ complex, which is a suitable receptor for the R-SNARE coming from the other fusion partner after mixing of the vesicles. However, similar reconstitution approaches with endosomal SNAREs yielded functional combinations of SNAREs in several different topologies [11], suggesting that different distributions of Q- and R-SNAREs over the two membranes can in principle form functional complexes. Studies with single SNARE molecules indicated even greater flexibility. For example, they showed the existence of anti-parallel associations of SNAREs. Thus, SNAREs can associate in multiple topologies [11]–[14].

An important question arising from these observations is whether in a cellular environment SNAREs have the liberty to associate in such variable topologies, or whether SNARE-associated tethering and docking proteins, such as the Rab-GTPases and their effector proteins, might control cis-SNARE disruption and guide subsequent SNARE association in a preferred topology *in trans*. Research on reconstituted, purified SNAREs has only recently begun to incorporate SNARE-associated proteins, such as SM-proteins, Rabs, and Rab-effectors into the reconstitutions [10],[15]–[16]. However, these co-reconstitutions have not yet been used to explore a possible effect of these components on the assembly pathway and topology of SNARE complexes. In physiological membranes, the issue of cis-SNARE complex disassembly and its transformation into trans-complexes has been studied on endosomes [17]. Endosomes contain the cognate set of endosomal SNAREs necessary for their fusion, as well as SNAREs for fusion at the plasma membranes. Cognate as well as non-cognate combinations of SNAREs could be co-precipitated from endosomal fractions, leading to the conclusion that there is promiscuity in cis-SNARE complex assembly.

Cis-SNARE complexes can accumulate as a product of a preceding fusion reaction or by spontaneous re-association of separated SNAREs *in cis*. They are reactivated by Sec18/NSF and its cofactor Sec17/α-SNAP [6],[18]. These chaperones are generally believed to completely disrupt SNARE complexes, liberating the individual SNARE for subsequent reassembly into *trans*-SNARE complexes [5],[19]. This reassembly and the ensuing fusion depend on Rab-GTPases and SM proteins [20]. These conserved factors can interact and cooperate with further compartment-specific factors, e.g. Rab effectors (tether factors) and lipids such as phosphatidyl-inositol-3-phosphate, Munc13, or complexin [21]. Addition of purified NSF completely disrupts complexes of purified SNAREs as well as SNARE complexes on isolated endosomal or vacuolar membranes [22]. Furthermore, the t-SNARE subunits SNAP25 and syntaxin1 reside in non-overlapping membrane patches in plasma membrane sheets of PC12 cells, suggesting that these two SNAREs remain spatially separated on this membrane [23].

We have used the cell-free fusion of purified vacuoles as a model reaction to follow the fate of cis-SNARE complexes and their conversion into trans-complexes [24]. Yeast vacuoles harbor five SNAREs that are necessary for their fusion [22]. The Q_a_ SNARE Vam3, the Q_b_ SNARE Vti1, the Q_c_ SNARE Vam7, and the R-SNARE Nyv1 form the *trans*-SNARE complexes required for fusion. An additional R-SNARE, Ykt6, can be associated with these SNAREs. However, Ykt6 was not found in *trans*-SNARE complexes [25] and a significant fraction of Ykt6 leaves the vacuolar membrane upon priming [26].

In this study, we took three approaches in order to investigate the transition from cis- into *trans*-SNARE complexes in vacuole fusion. First, we tested which subunits of the *trans*-SNARE complex are contributed by one or the other fusion partner. Second, we created inactive SNARE mutations in the two fusion partners in combinations that allowed us to test the functional significance of the observed SNARE association. And third, we analyzed priming, the activation of cis-SNARE complexes, asking whether activation of cis-SNARE complexes indeed leads to complete disruption of cis-SNARE complexes. We tested, whether incomplete dissociation of cis-SNARE complexes might prejudice the trans-association of SNAREs in a certain topology.

## Results

### Vacuolar SNAREs Pair *in trans* in a Preferred Q_bc_R-Q_a_ Topology

In the past years the topology of *trans*-SNARE formation mainly has been addressed by employing liposome fusion systems, in which recombinantly expressed SNAREs have been reconstituted and tested for fusion activity [4],. While most of these studies gave evidence for a preferred Q_abc_-R topology, others indicated an alternative possibility of *trans*-SNARE formation [11],[13]. Moreover, experiments conducted under more physiological conditions suggested a preferred Q_bc_R-Q_a_ *trans*-SNARE topology [27],[28]. To unravel these contradictory observations, we decided to investigate the topology of *trans*-SNARE formation in the vacuolar fusion system. To accommodate recent reports that oxidation might affect SNARE function [29],[30], we strictly decided to work in all following experiments under reducing conditions by adding DTT to fusion reactions and detergent extracts. Indeed, by using non-reducing SDS-PAGE, we noticed that some vacuolar proteins change their migration behavior, suggesting that oxidation might occur during fusion and detergent extraction (Figure S1).

We used differential tagging of vacuolar SNAREs to probe the topology of the *trans*-SNARE complexes formed during vacuole docking. In agreement with published observations [31], we noticed that tagging vacuolar SNAREs on their cytoplasmic N-terminus interferes with fusion activity (C. Peters, unpublished results). Consequently, we fused all tags to the C-termini, which for the membrane-anchored SNAREs are at the lumenal face of the vacuolar membrane. All tagged SNAREs were expressed from their authentic loci in the genome under the control of their native promoters, i.e., no non-tagged allele of the respective SNARE was left in these cells (Table S1). The tagged strains were viable and grew normally. The expression levels of the proteins on vacuoles isolated from the tagged strains were normal (Figure S2), and their fusion activities were also comparable to those of untagged vacuoles (Figure S3).

Vacuole docking depends on trans-complex formation between Vam3-Q_a_, Vti1-Q_b_, Vam7-Q_c_, and Nyv1-R [5],[19]. Vam3-Q_a_, Vti1-Q_b_, and Nyv1-R are integral membrane proteins, whereas Vam7-Q_c_ is anchored to vacuoles by the phosphatidylinositol-binding PX domain [32]. Both fusion partners carry the same set of SNAREs, but vacuoles from strains expressing differently tagged SNAREs can be mixed in vitro. Differential peptide tagging thus allows the investigation to distinguish cis-associations occurring within the same membrane from trans-associations between SNAREs originating from the apposed fusion partners. Starting a fusion reaction with ATP produces trans-associations, which lead to fusion and hence are converted into post-fusion cis-complexes. In order to prevent this conversion, it is desirable to block fusion at a late stage. We noted that after an initial incubation for 5 min at 27°C, subsequent cooling of the reaction to 7°C efficiently suppresses fusion; we used this simple technique to accumulate docked vacuoles (Figure S4A). We tested whether the vacuoles could prime and dock by two-stage incubations, exploiting the fact that completion of priming renders the further course of fusion resistant to antibodies to Sec18p, while completion of docking renders it resistant to anti-Ypt7 [33],[34].

We incubated vacuoles under fusion conditions at 27°C for a 5 min period with control buffer or antibodies to Sec18p and Ypt7p, respectively, in order to stop further priming and docking in the presence of ATP (Figure S4B). Then, the reaction continued either at 27°C or 7°C for 30 min. In the absence of inhibitors, vacuoles arrested at 7°C efficiently completed fusion during the second incubation at 27°C for 30 min. They also did so in the presence of anti-Ypt7p or anti-Sec18p during the second incubation, suggesting that the initial pre-incubation at 7°C had rendered them resistant and permitted completion of priming and docking. If those two inhibitors already were present during the first incubation, no significant fusion was observed (Figure S4B). This suggests that at 7°C, the reaction passes the priming and docking stages and arrests at a productive intermediate stage beyond docking. Fusion inhibition at 7°C was not due to decreased reporter maturation, since adding Triton X-100 to the vacuoles, allowing fusion-independent maturation of pro-ALP, did not result in significantly different ALP activities at 7°C and 27°C (Figure S4C).

**Figure 1:**
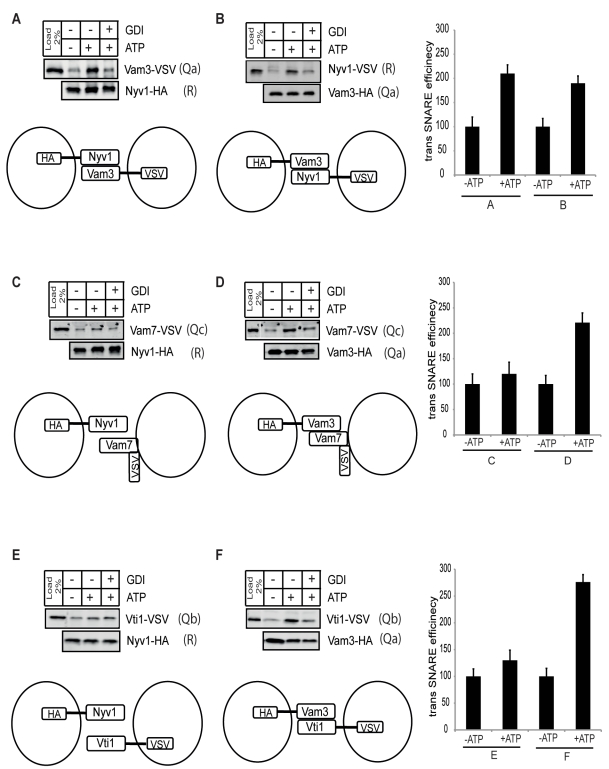
*trans*-SNARE complexes assayed by tagged SNAREs. Vacuoles were prepared from BJ3505 strains carrying individual SNAREs tagged with (HA)_3_ or VSV peptide epitopes at their C-termini. Vacuoles were incubated under standard fusion conditions in the presence or absence of ATP or in the presence of ATP and GDI. *Trans*-SNARE assay was performed as described under “Material and Methods.” After 30 min at 7°C, vacuoles were solubilized and immunoprecipitated with antibodies to the (HA)_3_ tag. Precipitated proteins were analyzed by SDS-PAGE and Western blotting against the indicated proteins. The −ATP value was set to 100%. The percentages of the +ATP and +ATP/GDI values were calculated. The quantification of protein band in Western blot was done by Odyssey densitometry. Three independent experiments are shown as means +/− SD.

We therefore used this 7°C incubation in order to accumulate *trans*-SNARE complexes and probe their topology (Figure 1). We mixed the Nyv1-HA(R) vacuoles either with Vam3-VSV(Q_a_), Vam7-VSV(Q_c_), or Vti1-VSV(Q_b_) vacuoles to test for a Q_bc_R-Q_a_ topology. We mixed Vam3-HA(Q_a_) vacuoles either with Nyv1-VSV(R), Vam7-VSV(Q_c_), or Vti1-VSV(Q_b_) to probe for a Q_abc_-R topology. After a 7°C incubation with ATP for 30 min, a time that is sufficient for complete docking [33],[34], the membranes were solubilized and immunoprecipitated against the HA tag. The degree of trans-association between the HA-tagged and VSV-tagged strains was assayed by Western blotting.

The observed results fell into two categories. The trans interactions among Vam3-Nyv1 (Q_a_-R), Vam3-Vam7 (Q_a_-Q_c_), and Vam3-Vti1 (Q_a_-Q_b_) increased from −ATP to +ATP fusion reaction. The increase was sensitive to the docking inhibitor GDI (Figure 1B, Figure 1D, and Figure 1F; Text S1). The trans interactions of Nyv1-Vam7 (R-Q_c_) and Nyv1-Vti1 (R-Q_b_) were comparatively much weaker than the Nyv1-Vam3 (R-Q_a_) or the Vam3-Vam7 (Q_a_-Q_c_) and Vam3-Vti1 (Q_a_-Q_b_) interactions (Figure 1A, Figure 1C, and Figure 1E). We also looked for homophilic interactions by tagging the same SNARE in both fusion partners with different tags, e.g., Vti1-HA (Q_b_) on one vacuole and Vti1-VSV (Q_b_) on the other. We could not detect any homophilic trans-interactions between Vti1-HA-Vti1-VSV (Q_b_-Q_b_), Vam3-HA-Vam3-VSV (Q_a_-Q_a_), or Nyv1-HA-Nyv1-VSV (R-R) (unpublished data). Thus, we did not obtain any indications that *trans*-SNARE complexes might multimerize.

In sum, our observations suggest that Nyv1-R, Vam7-Q_c_, and Vti1-Q_b_ are retained in a partial cis-SNARE complex that incorporates Vam3-Q_a_ from the other fusion partner. The resulting *trans*-SNARE complex hence should show a preferred Q_bc_R-Q_a_ topology, i.e., the Q_b_, Q_c_, and R-SNARE are predominantly contributed from the same membrane, whereas the syntaxin-like SNARE Q_a_ might act alone on the other fusion partner. It should be noted that we consider this as a preferred topology, since a certain amount of trans interactions between Nyv1-Vam7 (R-Q_c_) and Nyv1-Vti1 (R-Q_b_) can also be observed (Figure 1C and Figure 1E).

In principle these complexes can emerge not only from *trans*-SNARE pairing, but also from cis associations that might occur after fusion of the two differentially labeled vacuoles, or after solubilization of the membranes. In addition to the controls described above, two observations in the immunoprecipitation experiments argue against this and show that these SNARE complexes connected the apposed membranes before fusion. First, the efficiency of the coprecipitations was much lower if the membranes had been incubated in the absence of ATP, which prevents SNARE priming and fusion [5],[18]. Second, the Ypt7p inhibitor, GDI, reduced the associations. Thus, the trans associations depend on docking. These criteria argue in favor of the genuine existence of *trans*-SNARE complexes as displayed in Figure 1. Additionally, we excluded a random SNARE association occurring in the solubilizate by mixing primed detergent extracts of differently tagged versions of SNAREs, and found no random intermixing of these SNAREs into preexisting Q_bc_R complexes (Figure S5).

**Figure 2:**
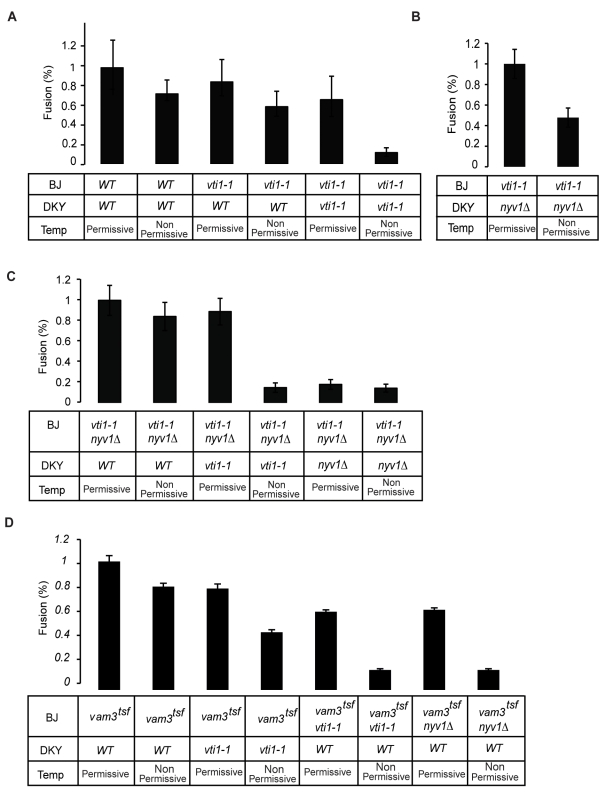
Inactivation of SNAREs by conditional alleles to create unidirectional *trans*-SNARE complexes. Vacuoles were isolated from BJ3505 (BJ) and DKY6281 (DKY) strains carrying the indicated temperature-sensitive alleles. The spheroplasting step of vacuole isolation was performed at 25°C (25 min, permissive) in order to leave the temperature-sensitive protein intact, or at 40°C (12.5 min for DKY, 25 min for BJ, non-permissive) to inactivate it. Isolated vacuoles were used in standard fusion reactions as indicated. Five independent experiments were averaged. Error bars indicate the standard deviation.

A Q_a_-Q_bc_R topology differs from the generally held Q_abc_-R model that a *trans*-SNARE complex assembles from a Q_abc_ SNARE subcomplex from one fusion partner and a single R-SNARE from the other fusion partner [3]. Therefore, we sought to test whether the Q_a_-Q_bc_R topology of the *trans*-SNARE complex corresponds to a functional restriction of SNARE requirements during vacuole fusion. To this end, we analyzed fusion reactions between vacuoles carrying combinations of SNARE mutations that allow the investigation to distinguish between the Q_abc_-R and Q_a_-Q_bc_R topologies (Figure 2). In an in vitro vacuole fusion assay, one can distinguish the two fusion partners because one vacuole type (BJ3505) contains a pro-alkaline phosphatase in the lumen, while the other contains the maturation enzyme (DKY6281). These two vacuole populations are separately prepared and mixed in vitro. Fusion between them generates mature alkaline phosphatase, whose activity serves to quantify fusion [24]. Despite the fact that the two fusion partners have different content, they have an identical pool of SNAREs in this homotypic fusion system. Topologically restricted *trans*-SNARE complexes can, hence, form in two orientations in a wildtype situation (Figure S6). Therefore, combinations of at least two mutations have to be distributed over the two fusion partners to circumvent this problem (Table S1). *Vam3-Q_a_*, *Vam7-Q_c_*, and *Nyv1-R* genes can be deleted without compromising viability. Since *Vti1-Q_b_* is essential, we used the conditional *vti1-1(Q_b_)* allele expressing a Vti1-Q_b_ protein that is inactivated at 40°C but remains functional at 25°C [35]. Cells can, therefore, be grown with functional Vti1-Q_b_. This Vti1-Q_b_ can then be inactivated by shifting the cells to 40°C during vacuole isolation (Figure 2A). If both fusion partners carried the *vti1-1* allele, fusion was blocked after pre-incubation at 40°C because neither fusion partner retains a functional Q_b_-SNARE, as shown earlier [22]. If only one fusion partner carried *vti1-1(Q_b_)*, but the other had the *wildtype* allele, fusion still proceeded efficiently, showing activities that were similar after preincubation at 40°C to those after preincubation at 25°C. After 40°C preincubation of the *WT/vti1-1(Q_b_)* combination, Vti1-Q_b_ remained functional only on the wildtype side (Figure 2A; Figure S6). This permits assembly of functional *trans*-SNARE complexes, but only in one orientation. In this situation we can ask which side contributes a certain SNARE subunit and functionally discriminate *trans*-SNARE topologies.

To discriminate between the different topology models, we deleted the *Nyv1-R* gene in one fusion partner and inserted the *vti1-1(Q_b_)* allele into the other. For this combination, the Q_abc_-R model predicts fusion because the *nyv1Δ-R* vacuole can provide a complete Q_abc_ t-SNARE and the *vti1-1(Q_b_)* vacuole can provide an R-SNARE. The Q_a_-Q_bc_R model, in contrast, predicts inhibition because neither fusion partner can provide the necessary Q_bc_R combination in one membrane (Figure S6). In the experiment *nyv1Δ-R* vacuoles fused with *vti1-1(Q_b_)* vacuoles after preincubation at 25°C, but displayed reduced fusion efficiency (60%) after preincubation at 40°C, which induces the mutant phenotype (Figure 2B; Figure S6). This result stands in support of a preferred Q_a_-Q_bc_R topology.

We created a second combination of mutations that allowed us to discriminate between the two models by mutating *Vti1-Q_b_* and *Nyv1-R* in the same membrane (Figure 2C; Figure S6). According to the Q_abc_-R hypothesis, such *vti1-1(Q_b_) nyv1Δ-R* vacuoles should not even fuse with a wildtype vacuole because they can neither provide a functional R-SNARE nor a functional Q_abc_-SNARE (Figure S6). The Q_a_-Q_bc_R model predicts fusion for this combination because the *vti1-1(Q_b_) Δnyv1-R* vacuole can provide Q_a_ SNARE, which can pair with Q_bc_R from the wildtype partner. We observed that the *vti1-1(Q_b_) nyv1Δ-R* mutant vacuoles fused almost equally well with wildtype vacuoles after pretreatment at 40°C or 25°C. One might invoke redundancy with other R-SNAREs to explain the remaining activity. This is unlikely, because only 10% residual activity remained when *vti1-1(Q_b_) nyv1Δ-R* vacuoles were incubated with fusion partners lacking functional *Nyv1-R or Vti1-Q_b_* (Figure 2C; Figure S6). Fusion between *vti1-1(Q_b_) nyv1Δ-R* and wildtype vacuoles, hence, depended on Vti1-Q_b_ and Nyv1-R, and did not result from substitution by another, non-vacuolar R-SNARE.

According to the Q_a_-Q_bc_R model, a vacuole containing an inactive Q_a_ and Q_b_ should be fusion incompetent, even in combination with a wildtype fusion partner. Such a double mutant has neither a functional Q_a_ nor a functional Q_bc_R combination (Figure S6). The Q_abc_-R model, in contrast, predicts fusion because the wildtype vacuole could provide a complete Q_abc_ SNARE and the Q_a_/Q_b_ double mutant still carries a functional R-SNARE. We tested this combination by inserting temperature sensitive *vti1-1(Q_b_)* and *vam3^tsf^-Q_a_* alleles [35],[36] into the same strain (Figure 2D; Figure S6). Although Vam3-Q_a_ is not essential, we had to use the *vam3^tsf^-Q_a_* allele because *vam3Δ-Q_a_* vacuoles also lack Vam7-Q_c_ and, hence, are not suitable for this type of analysis. The cells were grown at 25°C and subjected to a brief 40°C or 25°C treatment during the spheroplasting step of vacuole isolation. Isolated mutant vacuoles were then fused to wildtype vacuoles. In this situation, when only one of the two mutations was present, preincubation at 40°C hardly reduced fusion activity (20%) in comparison to preincubation at 25°C. A more severe effect was observable when the two mutations were distributed over the two fusion partners (reduction of fusion efficiency of about 50%). However, when *vti1-1(Q_b_)* and *vam3^tsf^-Q_a_* mutations were combined in the same vacuole, the severest fusion defects became apparent. Already at 25°C, the double mutant vacuoles retained only 50% of the activity of the single mutants. Upon brief pretreatment at 40°C, fusion was almost completely suppressed. This result is consistent with the Q_a_-Q_bc_R model.

To exclude a priming defect caused by the double SNARE mutation in the *vti1-1 (Q_b_) vam3^tsf^-Q_a_* mutant, we tested SNARE-complex stability by immunoprecipitation of Nyv1-R from detergent extracts of wildtype and mutant vacuoles incubated at restrictive temperature. While wildtype vacuoles showed the persistence of a Q_bc_R complex, mutant vacuoles displayed an unstable complex but were able to prime (Figure S7A). As a further control, we tested the influence of a *nyv1Δ-R* mutation in the *vam3^tsf^-Q_a_* background. As expected, this mutant fused as well as the single vam3^tsf^-Q_a_ at permissive temperature, but lost fusion activity at restrictive temperature due to inactivation of R and Q_a_ SNARE on the same membrane (Figure 2D; Figure S6). Additionally, we measured reporter loading of the different SNARE-mutants by incubating DKY and BJ vacuoles in the presence of Triton X-100. We found that in the presence of the *vam3^tsf^-Q_a_* mutation, BJ vacuoles contained only 50% of pro-ALP loading. We accommodated this by doubling the incubation time in developing buffer (Figure S7B).

### Post-Priming *Cis*-SNARE Complexes Are Predominantly Composed of Q_bc_R

Based on the observation that both biochemical and functional analysis revealed a preferred Q_bc_R-Q_a_ topology in vacuolar *trans*-SNARE formation, we asked whether a stabilization of a primed Vam7/Vti1/Nyv1 (Q_bc_R) complex *in cis* could prejudice the topology of the *trans*-SNARE complexes that form during subsequent docking.

Isolated vacuoles contain cis-SNARE complexes that are activated and disrupted by Sec18 upon addition of ATP [22]. We confirmed this result when working under similar conditions. However, as mentioned in the beginning of the Results section, we performed our fusions and immunoprecipitations in the presence of DTT and investigated how cis-SNARE complexes behaved under this condition. In order to monitor the assembly of cis-SNARE complexes, we first precipitated Vam3-Q_a_ from detergent extracts. Specifically, we were interested in determining whether a persistent post-priming Q_abc_ (Vam3-Q_a_, Vti1-Q_b_, Vam7-Q_c_) complex might be established after the NSF-mediated priming process, as predicted by the current model of *trans*-SNARE formation [3]. Although this Q_abc_-complex formation has been demonstrated for recombinant proteins and is routinely used in liposome fusion assays, it has not yet been detected on physiological membranes.

**Figure 3:**
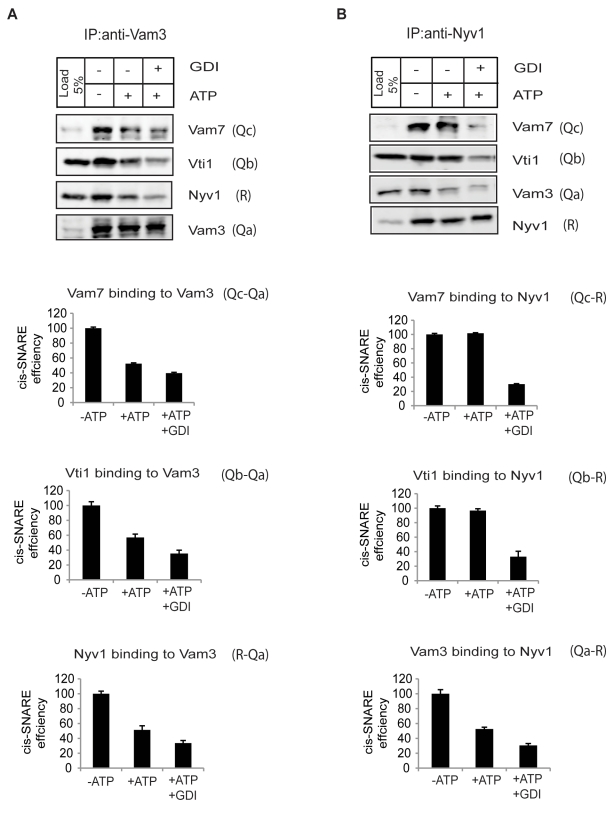
Q_bc_R-complex formation during the priming reaction. Vacuoles from wildtype cells were incubated under standard fusion conditions in the presence or absence of ATP and GDI. After 5 min of incubation at 27°C, vacuoles were solubilized with Triton X-100 and immunoadsorbed to anti-Vam3 (Figure 3A) or anti-Nyv1 (Figure 3B) protein A beads. Bound proteins were separated by SDS-PAGE, blotted, and probed with indicated antibodies. The efficiency of Q_abc_ complex formation was determined as co-precipitation of Vam7 and Vti1 with Vam3; the efficiency of Q_bc_R complex formation was determined as co-precipitation of Vam7 and Vti1 with Nyv1 in wildtype vacuoles and standardized to the −ATP amount. The percentages of the +ATP and +ATP/GDI values were calculated. The quantification of protein band in Western blot was done by Odyssey densitometry. Five independent experiments are shown as ± SD.

SNARE-activation was started by addition of ATP. Vacuoles that did not receive ATP could not activate their cis-SNARE complexes, and hence served as a negative control. After 5 min, EDTA was added in order to stop further hydrolysis of ATP. This short period of ATP exposure only allows cis-SNARE assembly, since *trans*-SNARE formation depends on docking and needs longer time to occur [24]. In vacuoles incubated without ATP, Vti1-Q_b_ and Vam7-Q_c_ co-fractionated with Vam3-Q_a_ as expected (Figure 3, left panel). Consistent with earlier experiments [5],[22] a substantial part of Vti1-Q_b_, Vam7-Q_c_, and Nyv1-R were released from Vam3-Q_a_ in the presence of ATP. Surprisingly, when Nyv1-R was precipitated from wildtype vacuoles, we did not observe this instability for a Q_bc_R complex (Figure 3, right panel). In contrast to Vam3-Q_a_, Vam7-Q_c_ and Vti1-Q_b_ remained tightly associated with Nyv1-R in the presence of ATP. We quantified the difference in persistence of Q_bc_R and Q_abc_ complexes in the presence of ATP (Figure 3). Vam3-Q_a_ lost about 50% of associations with all other SNAREs, whereas Nyv1-R only was separated from Vam3-Q_a_ (at a rate of about 50%) and retained association with Vam7-Q_c_ and Vti1-Q_b_ at an extent of almost 100%. We interpreted this result as a preferred generation of a stable post-priming Q_bc_R complex instead of an expected Q_abc_ complex, although this effect was not absolute, since also a substantial part of Q_abc_-complexes sustained ATP exposure.

Is maintenance of cis-SNARE associations relevant to the establishment of *trans*-SNARE complexes and to subsequent fusion? In order to address this question, we tested three different conditions that destabilize cis-SNARE interactions for their effect on *trans*-SNARE pairing and fusion.

**Figure 4:**
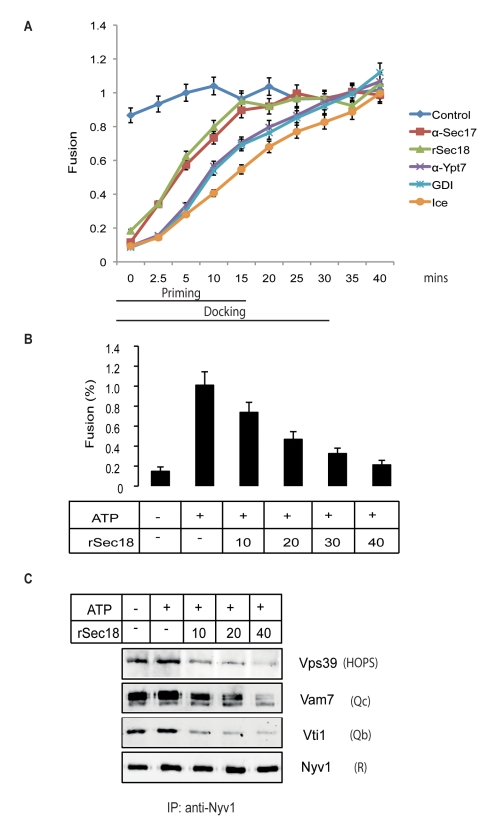
Excess rSec18 inhibition kinetically localizes to the priming reaction. (A) Kinetic analysis of rSec18 inhibition. Standard fusion reactions were started at 27°C. At the indicated times, inhibitors or control buffer were added. The samples were left on ice for 5 min. Then, they were transferred to 27°C or left on ice for the remainder of the 40 min reaction period. After 40 min, fusion activity was assayed. The following inhibitors were used: anti-Sec17 (3 µM), anti-Ypt7 (5 µM), and rSec18 (50 µg/ml). (B) Inhibition of vacuolar fusion by increasing amounts of rSec18 (µg/ml) added to standard fusion reactions. After 60 min of incubation at 27°C, vacuoles were assayed for fusion activity as described in experimental procedures. Three independent experiments are represented as means ± SD. See also Figure S3. (C) Increasing concentrations of rSec18 disassemble otherwise stable Q_bc_R-complexes and destabilize Q_bc_R-HOPS interactions. rSec18 was added at the indicated amounts (µg/ml) to vacuoles and incubated for 5 min at 27°C. Thereafter, vacuoles were detergent-extracted and Nyv1 was precipitated. Vps39 depicts the extent of HOPS-Q_bc_R-complex binding.

First, we used excess rSec18 (Text S1) as a tool to specifically destroy cis-SNARE complexes and correlated this with the inhibitory effect of excess rSec18 on vacuolar fusion during the priming phase. In vacuole fusion, priming (cis-SNARE activation) and docking (trans-complex formation) can be distinguished by determining the time point at which a fusion reaction becomes resistant to the addition of different inhibitors [18],[24]. We tested the effect of excess of Sec18/NSF on the priming or docking phase. We used antibodies to Sec17/α-SNAP, which inhibits priming (acts on priming phase of fusion curve, 0–15 min), and antibodies to the vacuolar Rab-GTPase Ypt7 or GDI, which inhibits docking (acts on docking phase of fusion curve, 0–30 min; Figure 4A). Numerous parallel fusion reactions were started. The inhibitors were added at different times after the onset of a fusion reaction. After addition of the inhibitor, the incubation was continued at 27°C until the end of the normal fusion period, and finally fusion activity was assayed. Control samples received only buffer before being re-transferred to 27°C, or they were set on ice in order to stop the reaction at this time point. The fusion reactions became resistant to excess rSec18/NSF after 15 min, with the same time course as to anti-Sec17/α-SNAP. Resistance to anti-Sec17/-αSNAP is a marker for the completion of priming. Resistance to anti-Ypt7 or GDI as markers for the completion of docking was attained after 30 min, the time at which the docking reaction is completed. This suggests that excess rSec18/NSF affects the priming phase of vacuole fusion but is not inhibiting docked vacuoles that have passed this stage.

Based on this observation, we investigated its influence on the stability of cis-SNARE complexes, whose existence locate to the same time period. The rationale of this experiment is that we tried to force a disassembly of reduced cis-SNARE complexes by adding an excess of purified rSec18/NSF to ATP-containing fusion reactions (Figure 4B), thereby gaining evidence for a fusion relevant role for these complexes. Indeed, increasing concentrations of rSec18/NSF gradually destabilized the association of Vti1-Q_b_ and Vam7-Q_c_ with Nyv1-R (Q_bc_R). This destabilization was not observed, even with the highest concentration of rSec18/NSF, when ATP was omitted from the incubation (unpublished data).

To monitor proper rSec18 activity, we subjected each Sec18 preparation to a quality control employing wildtype and *vtc4Δ* vacuole fusion reactions (Figure S8). The vacuolar Vtc-complex comprises multiple subunits and displays a polyphosphate synthase activity [37], which is for yet unknown reasons linked to Sec18 activity. Vacuoles purified from *vtc4Δ* strains strictly depend on the addition of functional rSec18 for their fusion activity since endogenous Sec18 function is impaired on these vacuoles [38]. Therefore, addition of rSec18 to vacuoles derived from *vtc4Δ* strains leads to stimulation of fusion at lower concentrations, but to inhibition of fusion at higher concentrations as observed for wildtype vacuoles (Figure S8).

As the addition of GDI led to the inhibition of *trans*-SNARE formation and destabilized cis-Q_bc_R complexes (Figure 1 and Figure 3), we speculated whether excess rSec18/NSF might influence the interaction of the Q_bc_R complex with the Ypt7-effector HOPS. HOPS is the tethering complex of vacuolar system composed of six different subunits, one of which is termed as Vps39 [20]. If the physical presence of HOPS is needed for stabilizing the post-priming Q_bc_R complex, excess Sec18/NSF might compete for or prevent the binding of the Q_bc_R complex to HOPS.

We therefore probed for the presence of Vps39 in the Nyv1-R-precipitations in the presence of increasing amounts of rSec18/NSF (Figure 4C). The concentration range in which Sec18/NSF destabilized the cis-SNARE associations led to a corresponding decrease in association with HOPS, indicating that HOPS and Sec18/NSF compete for binding to the Q_bc_R complex. Concomitantly, fusion activity of the vacuoles decreased with increasing concentrations of Sec18/NSF (Figure 4B). While this decrease of fusion activity correlates to the disassembly of the cis-SNARE interactions, it could also reflect the disassembling activity of Sec18/NSF on *trans*-SNAREs. This appears unlikely, since the kinetic analysis displayed in Figure 4A excludes a direct effect of Sec18/NSF on *trans*-SNARE complexes, suggesting that they are resistant to disassembly, consistent with the increased NSF resistance of *trans*-SNARE complexes observed in a liposome system [10],[39].

**Figure 5:**
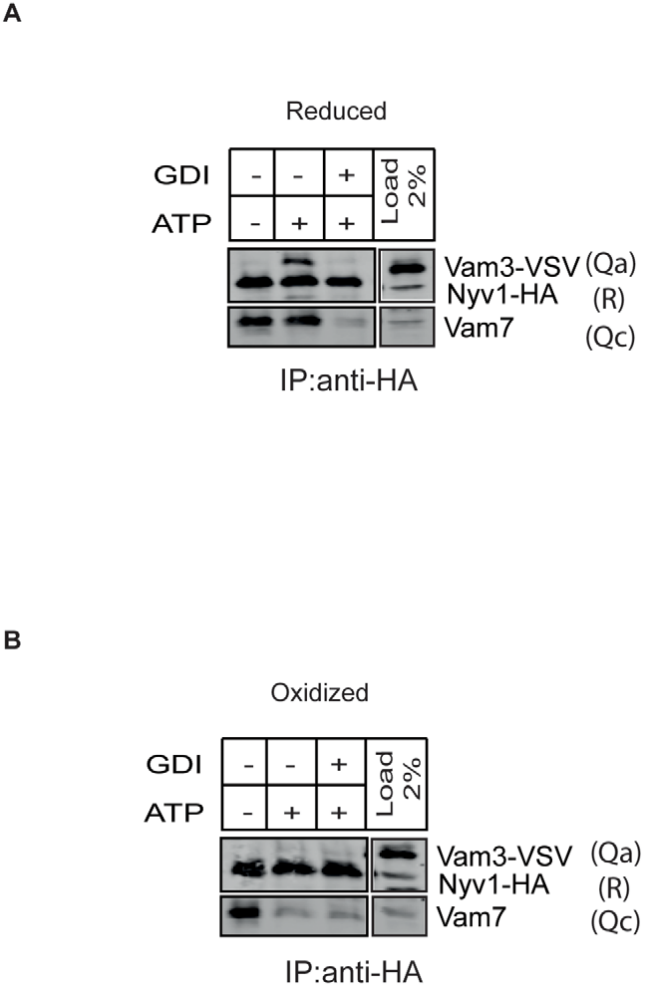
Effect of oxidation on *trans*-SNARE complex formation. *Trans*-SNARE assay for oxidized and reduced vacuoles. Reduced (Figure 5A) or oxidized (Figure 5B) Nyv1-HA and Vam3-VSV tagged vacuoles were harvested from gradients containing DTT or H_2_O_2_. Oxidized or reduced Nyv1-HA and Vam3-VSV vacuoles were mixed in a 1∶1 ratio and incubated in the presence or absence of ATP and GDI. After 5 min of incubation at 27°C and 30 min incubation at 7°C, vacuoles were solubilized with Triton X-100 in PS buffer and immunoadsorbed to anti-HA protein G beads. Bound proteins were separated by SDS-PAGE, blotted, and probed with the indicated antibodies. Oxidation does not impair priming, but suppresses trans-SNARE formation.

Second, we deliberately oxidized vacuoles and probed the stability of cis-SNARE complexes under this condition in order to investigate the consequence of unstable Q_bc_R -complexes for the following *trans*-SNARE establishment (Figure 5 and Figure S9). We tested this by mixing Nyv1-HA(R) vacuoles with Vam3-VSV(Q_a_) vacuoles. Mixing these two populations allows us to identify trans-interaction (Nyv1-HA/Vam3-VSV, R-Q_a_). After 30 min of incubation in the presence of ATP, trans-interactions increased significantly. These trans-interactions were sensitive to GDI, which inhibits the vacuolar Rab-GTPase, Ypt7p (Figure 5A), and thereby prevents tethering and docking [20]. In contrast, oxidized vacuoles did not form ATP-dependent *trans*-SNARE interactions (Figure 5B) even though the priming of the *cis*-SNARE complexes occurs normally, as evident from the ATP-dependent destabilization of the Nyv1-HA/Vam7 (R-Q_c_) interaction (Figure 5B).

Third, we inactivated Ypt7 by addition of GDI and asked whether this might influence *cis*-SNARE-stability and give evidence for the involvement of the tethering machinery in *cis*-SNARE complex stabilization. The fact that GDI is an effective inhibitor of *trans*-SNARE formation (Figures 1 and 5A) led us to speculate about a possible influence of this inhibitor on *cis*-SNARE stability. This is not evident from the kinetic analysis displayed in Figure 4A, as the inhibitory effect of GDI is clearly located on the docking curve. But this does not exclude that GDI might affect fusion components at an earlier stage of membrane fusion, since the architecture of the kinetic experiment shown in Figure 4A only resolves the latest fusion inhibitory effect of GDI. Moreover, the observation that excess rSec18 already inhibits the interaction of HOPS with the Q_bc_R-complex in the priming reaction (Figure 4C) points to a possible role of Ypt7 during an earlier phase of vacuolar fusion. Indeed, addition of GDI destabilizes the Q_bc_R–complex, indicating that HOPS and Ypt7 are required for the persistence of the Q_bc_R-complex during the priming phase of vacuolar fusion (Figure 3B).

Taken together, these findings suggest that destruction of this *cis*-SNARE association by excess Sec18/NSF, or by oxidation of the vacuoles, or by Rab-inactivation leads to inefficient *trans*-SNARE pairing and fusion deficiency.

### Stability of Post-Priming Q_bc_R Complexes Depends on the Presence of Rab-GTPase/HOPS and Their Effectors

**Figure 6:**
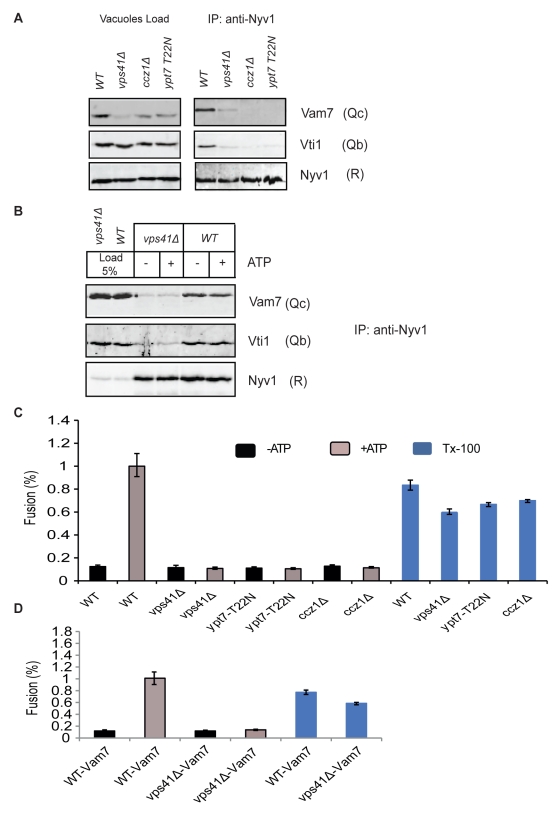
Q_bc_R-SNARE complexes on tether-mutant vacuoles. (A) Presence of Q_bc_R complexes on tether mutant vacuoles assayed by precipitating Nyv1. The left panel depicts expression levels of SNAREs on purified wildtype, *vps41Δ*, *ccz1Δ*, and *ypt7-T22N* vacuoles; the right panel shows precipitated QQR-complexes from these vacuoles in the absence of ATP. (B) Vam7-overexpression on *vps41Δ* vacuoles does not restore Q_bc_R-complex formation. Vam7 was over-expressed in wildtype and *vps41Δ* strains, and Q_bc_R-complex formation was assayed by precipitating Nyv1 in the presence and absence of ATP. (C) Fusion rates of wildtype and tether-mutant vacuoles. Vacuoles were fused under standard fusion conditions. TX-100 values depict ALP maturation of mixed vacuoles in the presence of 0.2% Triton X-100 and therefore show the amount of reporter loading. Three independent experiments are displayed as means +/− SD. (D) Fusion rates of wildtype and *vps41Δ* vacuoles over-expressing Vam7. Vacuoles were fused under standard fusion conditions. Three independent experiments are shown as means +/− SD.

To further confirm that members of the tethering machinery are indispensable for stabilizing a post-priming Q_bc_R-complex, we tested the dependence of *cis*-SNARE pairing on the Rab-GTPase, Ypt7, and its GEF, the Ccz1/Mon1 complex ([40]–[42]; Figure 6), and on the Ypt7 effector complex subunit Vps41p [43],[44]. We assayed the existence of the Vam7/Vti1/Nyv1 (Q_bc_R) association in *Δvps41* vacuoles, in *ccz1Δ* vacuoles, and in *ypt7* vacuoles expressing the T22N allele of Ypt7, which produces Ypt7 protein mimicking the GDP-bound state [45]. Since the *vps41Δ* mutant showed significantly reduced Vam7-Q_c_ levels on the vacuoles (Figure 6A), but not in the whole cell (Figure S10), we corrected this deficiency by over-expressing Vam7-Q_c_, yielding mutant vacuoles that were similar to wildtype vacuoles (Figure 6B). Despite almost normal wildtype expression levels of Vam7-Q_c_, mutant vacuoles showed strongly reduced *cis*-SNARE association of Nyv1-R, Vam7-Q_c_, and Vti1-Q_b_ (Figure 6A and Figure 6B). Upon addition of ATP, these strongly reduced levels did not decrease further, suggesting that they represent the background of the assay. The absence of functional *cis*-SNARE complexes in all mutants leads to fusion-incompetent vacuoles (Figure 6C and Figure 6D). These results might explain earlier findings [42],[43] and support the notion that the stabilization of a *cis*-SNARE complex of Nyv1-R, Vam7-Q_c_, and Vti1-Q_b_ depends on the GTP-bound form of the Rab-GTPase Ypt7 and on the presence of a functional HOPS complex.

### The Stability of the Q_bc_R Complex Does Not Depend on *Trans*-SNARE Interactions

**Figure 7:**
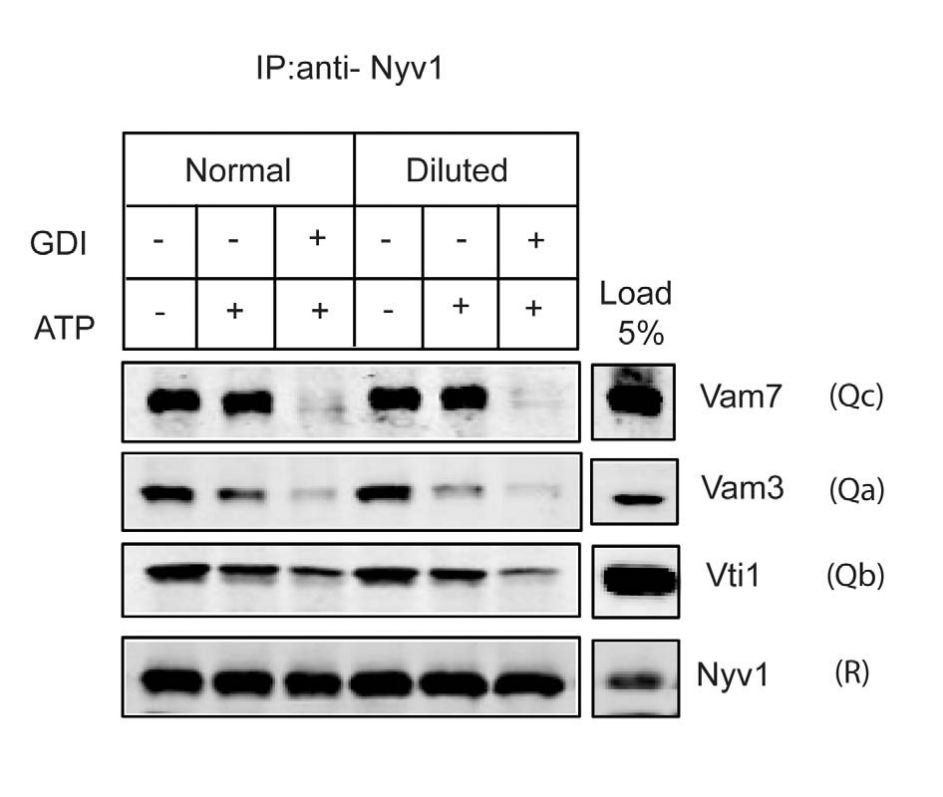
The Q_bc_R-complex stability does not depend on trans-SNARE interactions. Isolated vacuoles were either fused under standard condition or diluted up to a density of 100 µg/ml prior to the addition of salt and ATP. After incubation for 5 min at 27°C, the standard fusion reaction (500 µg/ml) was diluted to the same density of 100 µg/ml, and 3 mM EDTA was added. After addition of Triton X-100 to a final concentration of 0.5% and brief centrifugation, Nyv1 was precipitated as described above. We found no difference in Q_bc_R-complex stability between diluted and non-diluted vacuoles.

The involvement of the tethering factors Ypt7 and HOPS in post-priming SNARE-complex stabilization presents an implication that *trans*-SNARE interactions may help to generate these SNARE complexes. In order to exclude this possibility, and to clearly demonstrate that Ypt7 and HOPS act *in cis* to stabilize post-priming SNARE complexes, we performed dilution experiments. Prior to the addition of salt, isolated vacuoles were diluted up to a density that does not support fusion. This is an indication that contact is lacking between vacuoles and, therefore, establishment of *trans*-SNARE interactions is not to be expected. We found no difference in Q_bc_R complex stability between vacuoles fused under standard conditions and diluted vacuoles (Figure 7), clearly demonstrating that Ypt7 and HOPS act in cis to stabilize the post-priming Q_bc_R-complex.

## Discussion

**Figure 8:**
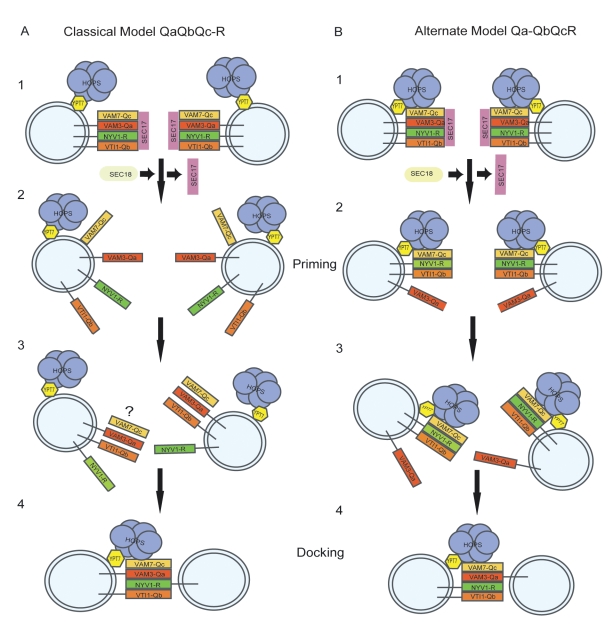
Different models for priming and docking. (A) According to the current model, SNAREs are totally separated by Sec18 (NSF) in the presence of ATP. Three Q-SNAREs reassemble to an acceptor complex probably catalyzed by tethering factors. The question mark indicates the fact that a trimeric Q-SNARE complex has not yet been identified on a physiological membrane. Finally, SNAREs dock and zipper up in a Q_abc_-R topology. (B) The alternate model comprises an activation step by Sec18 leading to a removal of the Q_a_-SNARE Vam3 and a persisting Q_bc_R-complex stabilized by HOPS/Ypt7. Subsequently, the SNAREs dock and zipper up in a Q_bc_R-Q_a_ topology.

The assembly pathway for *trans*-SNARE complexes and the resulting topology are of fundamental importance for the control of fusion reactions. The fact that Rab-GTPases and tether proteins must stabilize subcomplexes of SNAREs will determine whether these proteins must act on vesicles or target membranes and will determine the possibilities for control of fusion reactions by signaling cascades. Control by external signals can only be studied once the assembly process has been elucidated. The final topology of the assembled *trans*-SNARE complex, i.e., the distribution of its subunits over the two membranes, should also influence its activity. SNARE subunits are membrane-anchored, in most cases by transmembrane helices and in few others by lipidation or by lipid binding domains [46]. It has been proposed that the orientation of the SNARE complex should influence its capacity to exert stress on the membrane, disturb the bilayer structure, and induce fusion [47]. Whether a given subunit of the *trans*-SNARE complex is anchored in one fusion partner or the other must determine the rotational orientation of the complex between the two membranes (see model in Figure 8). Since the SNARE complex itself is of considerable size—and hence an obstacle to direct contact between the lipid bilayers [48], its twisting could induce strong local strain on the bilayer, using the large hydrophilic part of the complex as a lever. Therefore, it appears likely that the assembly and situation of the trans complex are restricted and controlled by cells.

Studies with purified SNAREs, both in soluble or liposome-associated form, indicated that the Q_a_, Q_b_, and Q_c_ helices spontaneously preassemble in the target membrane in order to form a Q_abc_ acceptor complex for an R-SNARE from the other fusion partner [49]–[50]. Our studies and the results from ER-Golgi transport and regulated exocytosis suggest, however, that in intact membranes, SNARE complex assembly occurs via a Rab- and tether-stabilized QbcR subcomplex. These discrepancies probably reflect the absence of constraints for SNARE assembly in the liposome systems, constraints that are imposed in the intact membrane system by Rab-GTPases and tether factors. Purified single SNAREs are largely unstructured [49]–[51]. Their rearrangement into a coiled-coil conformation can be kinetically limiting for *trans*-SNARE complex formation and fusion of proteoliposomes. In the absence of other factors, Q_a_, Q_b_, and Q_c_ helices can form stable subcomplexes that can subsequently integrate an R helix. Therefore, co-reconstitution of a SNARE combination that allows slow pre-structuring of a *cis*-SNARE subcomplex in one membrane (e.g., during production and purification of the proteoliposomes) can render the integration of the remaining SNARE helix from the other fusion partner much faster and strongly enhance the rate of fusion. This explains why in some studies, proteoliposomes in which Q_a_, Q_b_, and Q_c_ SNAREs were co-reconstituted into one vesicle and the R-SNARE in the other yielded higher fusion activities [4]. Depending upon the experimental condition chosen, however, other distributions of the four SNAREs over the two membranes can become fusogenic [11]. This important result illustrates that SNAREs can assemble into trans complexes in various topologies. In a physiological membrane, by contrast, SNAREs are associated with Rab and tether proteins that may restrict the assembly pathway. These control factors recently were shown to further stimulate fusion of SNARE-containing liposomes [52],[53], but how they influence the assembly pathway and topology of *trans*-SNARE complexes in these reactions has not yet been resolved.

Our results suggest that Sec18/NSF selectively removes the Q_a_ SNARE from vacuolar *cis*-SNARE complexes, generating a Q_bc_R subcomplex that is stabilized by the Rab-GTPase Ypt7 and the associated HOPS complex. This Q_bc_R subcomplex serves as a template for integrating a Q_a_ SNARE from the other fusion partner.

Two possibilities of HOPS mediated *cis*-SNARE stabilization are conceivable. Either HOPS stabilizes a partially zippered up Q_bc_R complex or single SNAREs are separately coordinated on HOPS subunits. Although we cannot say how exactly HOPS stabilizes an intermediate Q_bc_R complex, it is evident that Ypt7 in the GDP-bound state does not scaffold a Q_bc_R cis complex, suggesting that active control via Ypt7 occurs. The functional relevance of this topology is supported by the observation that vacuoles retain their fusion competence only if inactivating SNARE mutations are distributed over the two fusion partners in combinations permitting formation of a Q_bc_R-Q_a_ trans-complex. We noted that adding an excess of the Q_c_-SNARE Vam7 permits the production of large quantities of Q_abc_-R complexes also in the vacuole system (C. Peters and A. Mayer, unpublished). Excess Vam7-Q_c_ provides a fusion activity with reduced sensitivity to the Ypt7 inhibitor GDI [54], suggesting that excess Vam7-Q_c_ partially compensates for the lack of Ypt7 activity. *Trans*-SNARE complexes accumulated 3–5 times higher amounts than normal, but now mainly in a Q_abc_-R topology (unpublished data). This underscores the potential for forming Q_abc_-R trans-complexes—consistent with the liposome studies that used these combinations—but demonstrates that active Ypt7 and HOPS channel trans-complex assembly mainly into the Q_bc_R-Qa arrangement by restricting complete *cis*-SNARE disassembly.

Our approach and experimental system impose two limitations that raise caveats for this interpretation. First, only a small percentage of the SNAREs enter *trans*-SNARE complexes, which renders it impossible to firmly exclude the existence of *trans*-SNARE complexes in topologies other than Q_bc_R-Q_a_. Second, the persistence of *cis*-SNARE associations and the preference for the Q_bc_R-Q_a_ trans-associations are not absolute. Our interpretation of the interactions in the trans-complex, therefore, relies mainly on the observed trends and the increase in the abundance of these trans complexes. However, the significance of the observed Q_bc_R-Q_a_ interaction is supported by the functional effects of SNARE mutations distributed over the two fusion partners in combinations that allow one to distinguish between Q_bc_R-Q_a_ and Q_abc_-R trans-complexes. We consider this correlation to be a strong argument for the validity and relevance of the observed interactions.

Could the Rab-controlled assembly of *trans*-SNARE complexes in a Q_bc_R-Q_a_ topology also apply to other SNARE-dependent fusion systems? Several published observations suggest that this may be the case. For fusion of ER-derived COPII vesicles with the Golgi, the use of different combinations of temperature-sensitive SNARE proteins showed that the SNAREs Bos1-Q_b_ and Bet1-Q_c_ act on the vesicles, and only the SNARE Sed5-Q_a_ acts on the acceptor membrane [27],[55],[56]. Chemical depletion of the vesicular Sec22-R pool reduced fusion [57]. Bos1-Q_b_ interacts with Bet1-Q_c_ and also with the R-SNARE Sec22, and this latter interaction depends on the Rab-protein Ypt1 [58]–[60]. It is unknown whether these interactions represent pre- or post-fusion states, whether they change in the course of fusion, or whether they might have occurred after solubilization of the membranes. However, these findings fit seamlessly with the sequence of events that we resolved in vacuole fusion.

Furthermore, functional studies on regulated exocytosis in cracked PC12 cells favored a Q_bc_R receptor complex as a post-priming intermediate rather than a Q_abc_ complex. Scheller and colleagues showed that SNARE priming sensitizes exocytosis selectively to competition by soluble syntaxin (Q_a_) peptides, but not to VAMP2 (R) peptides [28]. Analyses of neurotoxin sensitivity at different stages of exocytosis further supported the hypothesis that priming might create a SNAP25/VAMP2 complex (Q_bc_R) that subsequently incorporates syntaxin (Q_a_) from the plasma membrane. Although *cis*- and *trans*-SNARE complex formations were not directly assayed in these studies, their results are compatible with our observations.

While functional studies on exocytosis in PC12 cells are consistent with a Q_a_ binding site being created by SNAP-25 (providing a Q_b_ and a Q_c_ helix) and VAMP2 (R), the major pool of SNAP-25 is located on the plasma membrane, whereas VAMP2 is mainly on the vesicles [61],[62]. In order to resolve this contradiction we can invoke two scenarios for formation of a VAMP2/SNAP-25 receptor complex [28]. One scenario is that SNAP-25 from the plasma membrane would first assemble with VAMP2 from the vesicle, creating a Q_bc_R complex that connects the two membranes. Alternatively, SNAP-25 on the vesicle could associate with VAMP2, creating a Q_bc_R cis complex. This latter scenario is supported by several studies that detected microscopically localized SNAP-25 on various types of secretory vesicles [23],[63] and provided convincing biochemical evidence for the presence of SNAP-25 in SNARE complexes on highly purified synaptic vesicles [64],[65]. Despite all these observations, we cannot rule out the existence of an alternative SNARE topology mediating neurotransmitter release. However, the combined evidence strongly favors the view that in physiological membranes, the Rab-GTPase and its associated tether factors bias *trans*-SNARE assembly by stabilizing a Q_bc_R receptor complex that integrates Q_a_ SNARE in a second step.

## Materials and Methods

### Vacuole Isolation

BJ3505 strains carrying tagged SNAREs were grown in YPD at 30°C at 225 rpm to OD_600_ = 2 and harvested (3 min, 5,000× g). Vacuoles were isolated as described [47], but all solutions contained 2 mM DTT and cell walls were hydrolyzed by lyticase [66], recombinantly expressed in *E. coli* RSB805 (provided Dr. Randy Schekman, Berkeley), and prepared from a periplasmic supernatant. Harvested cells were resuspended in reduction buffer (30 mM Tris/Cl pH 8.9, 10 mM DTT) and incubated for 5 min at 30°C. After harvesting as described above cells were resuspended in 15 ml digestion buffer (600 mM sorbitol, 50 mM K-phosphate pH 7.5 in YP medium with 0.2% glucose and 0.1 mg/ml lyticase preparation). After 20 min at 30°C, cells were centrifuged (1 min 5,800 rpm in JLA25.5 rotor). The spheroplasts were resuspended in 2.5 ml 15% Ficoll-400 in PS buffer (10 mM PIPES/KOH pH 6.8, 200 mM sorbitol) and 200 µl DEAE-Dextran (0.4 mg/ml in PS). After 90 s of incubation at 30°C, the cells were transferred to SW41 tubes and overlaid with steps of 8%, 4%, and 0% Ficoll-400 in PS. Cells were centrifuged for 60–75 min at 2°C and 30,000 rpm in a SW41 rotor. Cytosol was prepared as described [67].

### *Cis*-SNARE Assays

Nyv1 and Vam3 were precipitated from samples containing 1 ml of vacuoles at a concentration of 500 µg/ml. Vacuoles were primed for 5 min in PS buffer with 125 mM KCl, 0.5 mM MnCl_2_, 1 mM DTT, and ATP-regenerating system. GDI was added at a concentration of 5 µM. Prior to centrifugation, 3 mM EDTA was added and incubated for 15 min at 27°C. Vacuoles were centrifuged for 2 min at 20,000 g and solubilized in PS buffer supplemented with 50 mM KCl, 3 mM EDTA, 0.5% Triton X-100, and 3 mM DTT. After centrifugation (4 min, 20,000 g at 4°C), 15 µg of polyclonal antibodies and 50 µl of a 1∶1 slurry of protein A were added and gently rotated for 1 h at 4°C. The beads were subsequently washed three times with extraction buffer diluted 1∶1 with PS-buffer and subjected to SDS-PAGE and Western blotting. For dilution experiments vacuoles were harvested and adjusted to a density of 100 µg/ml prior to the addition of KCl. After incubation for 5 min at 27°C, 3 mM EDTA was added and further incubated for 15 min at 27°C. Subsequently, vacuoles were directly detergent extracted by adding Triton X-100 to a final concentration of 0.5%. After centrifugation the samples were processed as described above.

### *Trans*-SNARE Assays

Vacuoles were adjusted to a protein concentration of 500 µg/ml. The total volume of one assay was 1 ml containing equal amounts of the two fusion partners in PS buffer with 125 mM KCl, 0.5 mM MnCl_2_, and 1 mM DTT. Mixed vacuoles were incubated for 5 min at 27°C in the absence of ATP. The fusion reaction was started by adding ATP-regenerating system (0.25 mg/ml creatine kinase, 20 mM creatine phosphate, 500 µM ATP, 500 µM MgCl_2_). After 5 min at 27°C, the vacuoles were cooled down to 7°C and incubated further for 30 min at this temperature. Thereafter, 3 mM EDTA was added and vacuoles were centrifuged for 2 min at 4°C at 20,000 g. The pellet was resuspended in 1.5 ml solubilization buffer (0.5% Triton, 50 mM KCl, 3 mM EDTA, 3 mM DTT in PS). After centrifugation for 4 min at 4°C (20,000 g), the supernatant was incubated with 30 µl Protein G-beads (Roche) and 15 µg HA-antibodies (Covance, mouse monoclonal) for 1 h at 4°C with gentle shaking. The Protein G-beads were washed three times with 50 mM KCl, 0.25% Triton, 3 mM DTT, and 3 mM EDTA in PS buffer, and incubated for 5 min at 60°C in 2– concentrated reducing SDS sample buffer.

### Vacuole Fusion

DKY6281 and BJ3505 vacuoles were adjusted to a protein concentration of 500 µg/ml and incubated in a volume of 30 µl PS buffer (10 mM PIPES/KOH pH 6.8, 200 mM sorbitol) with 125 mM KCl, 0.5 mM MnCl_2_, 1 mM DTT. Inhibitors were added before starting the fusion by addition of the ATP-regenerating system (0.25 mg/ml creatine kinase, 20 mM creatine phosphate, 500 µM ATP, 500 µM MgCl_2_). After 60 min at 27°C, or on ice, 1 ml of PS buffer was added, vacuoles were centrifuged (2 min, 20,000× g, 4°C) and resuspended in 500 µl developing buffer (10 mM MgCl_2_, 0.2% TX-100, 250 mM TrisHCl pH 8.9, 1 mM p-nitrophenylphosphate). After 5 min at 27°C, the reactions were stopped with 500 µl 1 M Glycin pH 11.5 and the OD was measured at 400 nm.

### Preparation and Fusion of Conditional Mutants

For experiments implicating temperature-sensitive mutants, cells were grown in YPD at 25° and 225 rpm. Cells were harvested at OD_600_ = 2 and vacuoles were prepared essentially as described above, with the following modifications: Cells were incubated for 7.5 min with reduction buffer at 25°C. After centrifugation, pep4 cells were spheroplasted (25 min at 25°C or 40°C) in spheroplasting buffer containing 2 mM DTT. For *pho8Δ* cells, the spheroplasting step was performed for 12.5 min at 40°C or for 25 min at 25°C. If spheroplasting was performed at 40°C, the amount of lyticase was reduced by 50% compared to spheroplasting at 25°C. All further steps were as described above in solutions containing 2 mM DTT. Vacuoles from BJ3505 expressing a *vam3^tsf^* allele contained 50% less of the reporter enzyme pro-alkaline phosphatase. This was taken into account and corrected in calculating the fusion activities.

## Supporting Information

Figure S1 Effect of oxidation on mobility of SNAREs on SDS-PAGE, HOPS-SNARE binding, and fusion. (A) Oxidized or normal vacuoles were harvested from step gradients in the presence or absence of 0.03% (v/v) H_2_O_2_. An equal amount of vacuoles was directly mixed with SDS sample buffer with (right panel) or without (left panel) DTT. Another sample of equal amount of normal vacuoles was solubilized with Triton X-100 for 1 h at 4°C. Subsequently, a sample of the solubilizate was mixed with SDS buffer with or without DTT. These samples were separated by SDS-PAGE, blotted, and probed with the indicated antibodies. When run on a non-reducing SDS-gel, Nyv1, Vam3, and Sec17 displayed multiple bands (Figure S1A, left panel) depending on the time point at which vacuoles were picked. Vacuoles taken freshly from the ficoll gradient showed little or no oxidation, whereas vacuoles deliberately oxidized during centrifugation by adding H_2_O_2_, or samples taken from the solubilizate, contained significant amounts of oxidized proteins. In contrast to Vti1 and Vam7, the three aforementioned proteins comprise multiple cysteines and are therefore prone to oxidation. The multiple band patterns is not due to proteolysis since the presence of DTT in the SDS-sample buffer leads to the occurrence of only one single band for each displayed protein (Figure S1A, right panel). Due to this observation and the fact that the cytosol normally represents a reducing environment, we explored the influence of the redox conditions on SNARE behavior and fusion activity. (B) The cell-free fusion of yeast vacuoles is traced via maturation of the pro-alkaline phosphatase pro-Pho8p in one fusion partner by the maturase Pep4p contained in the other fusion partner. In order to create defined conditions before the start of fusion, we oxidized vacuoles by inclusion of 0.03% H_2_O_2_ (v/v) into the buffers for vacuole isolation, and we also prepared reduced vacuoles by including 1 mM DTT. The H_2_O_2_ concentration was chosen based on the observation that under this condition the fusion activity of vacuoles decreased to almost background level, but oxidation was reversible and did not lead to non-specific damage to the vacuoles. Whereas the reduced vacuoles were active for fusion, the oxidized vacuoles did not fuse (Figure S1B). Oxidized organelles could be reactivated for fusion by including 1 mM DTT in the fusion buffer. Addition of rVam7 (100 µg/ml), which is an elegant method to circumvent NSF-mediated priming [1], did not restore fusion activity of the oxidized vacuoles (Figure S1B, lane 6), indicating that the fusion defect of oxidized vacuoles is not due to inhibition of priming. Three independent experiments are shown as means ± SD. (C) Maturation of reporter was assayed in the presence of 0.2% Triton X-100 for reduced and oxidized vacuoles. 60 µg of vacuoles were incubated for 1 h at 27°C. Inhibition of fusion by oxidation was not due to an inactivation of the reporter system either because oxidized and reduced vacuoles gave similar alkaline phosphatase activity if the fusion sample was solubilized in Triton X-100, a treatment permitting fusion-independent access of Pep4p to pro-Pho8p. (D) Vacuoles harboring Vps33-HA as part of the HOPS complex were harvested from step gradients in the presence or absence of 0.03% (v/v) H_2_O_2_. After 15 min of incubation at 27°C in the absence or presence of ATP, vacuoles were detergent extracted and Vps33-HA was precipitated using protein G-absorbed antibodies. Bound proteins were separated by SDS-PAGE, blotted, and probed with the indicated antibodies. On oxidized vacuoles Vam7-Qc as part of the tethering-complex stabilized QQR-complex is no longer able to bind to the HOPS complex.
(TIF)

Figure S2 Expression rates of tagged SNAREs. For each tagged SNARE version, 30 µg of vacuoles were loaded on a SDS-PAGE followed by Western blotting with indicated antibodies against SNAREs. The added tags resulted in different running velocities of the SNARE proteins.
(TIF)

Figure S3 Fusion rates of vacuoles harboring tagged SNARE-versions. Fusion rates of vacuoles harboring tagged SNARE versions were measured by the standard hemifusion assay [2] and vacuole fusion assay. In both experiments, fusion rates of wildtype and tagged SNARE vacuoles were comparable.
(TIF)

Figure S4 Temperature dependence of fusion stages and fusion rates. (A) Temperature titration of standard vacuolar fusion reactions. Content mixing almost stops completely at 7°C. All samples were primed for 5 min at 27°C prior to the incubation at lower temperatures. (B) Incubation of vacuoles at 7°C for 30 min allows completion of docking but prevents content mixing. Vacuoles were incubated with (Lane 2&3) or without inhibitor for 5 min at 27°C (priming). Then the incubation was continued at 27°C (Lanes 1–3) or at 7°C (Lanes 4–7) for 30 min in the presence or absence of inhibitors (docking). Thereafter, inhibitors were added to Lanes 6 and 7. Subsequently all samples, except those of the 7°C control (lane 4), were shifted to 27°C and further incubated for 30 min (Fusion). (C) Processing of ALP is not dramatically affected by lowering temperatures. Vacuoles were either incubated for 30 min under standard fusion conditions at 27°C or 7°C in order to assay fusion or for 1 h at 27°C or 7°C in the presence of 0.2% Triton X-100 in order to assay reporter maturation.
(TIF)

Figure S5 SNAREs do not reassemble randomly into new complexes in the detergent extract. Vacuoles from strains harboring Nyv1-HA, Vti1-VSV, and Vam7-VSV were purified and primed under standard conditions. After separate solubilization, Nyv1-HA was precipitated either from samples containing only Nyv1-HA (100%) or from a detergent extract containing a mixture of Nyv1-HA, Vam7-VSV, and Vti1-VSV (50%, 25%, 25%). We did not observe any intermixing of Vam7-VSV or VTI1-VSV with Nyv1-HA in the detergent extract, indicating the stability of the vacuolar Q_bc_R-complex.
(TIF)

Figure S6 Combinations of v-SNARE deletions and conditional t-SNARE alleles to distinguish the Q_a_-Q_bc_R and Q_abc_-R topologies. Vacuoles were isolated from strains carrying the indicated combinations of deletions or temperature-sensitive alleles. The table depicts a comparison of observed fusion effects with the predictions by the Q_a_-Q_bc_R and Q_abc_-R models. Green colored SNARE combinations show fusion; red colored SNARE combinations show no fusion.
(TIF)

Figure S7 The *vti1-1 vam3^tsf^* double mutant primes normally, but displays an unstable Q_bc_R-complex. (A) Vacuoles from wildtype and *vti1-1 vam3^tsf^* mutant cells were purified under non-permissive conditions and Q_bc_R-complex stability was assayed as described above. Wildtype vacuoles displayed the expected stable Q_bc_R-complex, whereas vacuoles derived from the double mutant showed loss of most of the Vam7 and Vti1 from Nyv1, indicating that priming works but post-priming Q_bc_R-complex stability is lost. (B) Reporter control for different SNARE^ts^ mutants: BJ and DKY vacuoles were purified from the indicated strains and incubated for 60 min at 27°C in the presence of 0.2% TritonX-100. All BJ mutants harboring the *vam3^tsf^* mutation showed only 50% ALP loading compared to wildtype vacuoles, which was taken into account by a longer incubation time in developing buffer.
(TIF)

Figure S8 rSec18 influence on vacuolar fusion at different concentrations. Vacuoles from wildtype cells (BJ&DKY) or *vtc4Δ* (BJ&DKY) were incubated under fusion conditions in the presence or absence of ATP and rSec18. Recombinant Sec18 was added at increasing concentration ranging from 1 µg/ml up to 40 µg/ml. As a buffer control, rSec18 samples were heat inactivated, and the remaining supernatant added to the fusion reaction with the same volume as active rSec18. After 60 min of incubation at 27°C fusion, activity was assayed as described in “Materials and Methods.”
(TIF)

Figure S9 Influence of oxidation on the persisting Q_bc_R-complex. (A) Nyv1 was precipitated from oxidized and reduced vacuoles or from vacuoles that were oxidized during the isolation procedure but were complemented with DTT in the fusion reaction. In the absence of ATP, Vti1 and Vam7 co-fractionated with Nyv1 under all conditions. ATP addition efficiently separated Vam7 and Vti1 from Nyv1 for the oxidized sample, consistent with published observations. However, the association of Vam7 and Vti1 with Nyv1 persisted even after ATP incubation for the reduced sample. Oxidized vacuoles that had been primed under reducing conditions behaved in a similar manner as those that were kept under reducing conditions throughout. This suggests that the destabilization of *cis*-SNARE complexes by oxidation of vacuoles is reversible. (B) Oxidation of proteins mainly occurs in the detergent extract. Vacuoles prepared in the absence of DTT fuse almost as efficiently as vacuoles prepared in its presence (Figure S1B). Therefore, we asked whether, in the absence of deliberate oxidation of the vacuoles by H_2_O_2_, the *cis*-SNARE complexes might remain stable enough during priming and docking, but decay subsequently in the solubilizate. Oxidized and reduced vacuoles were harvested from gradients containing H_2_O_2_ or DTT. Vacuoles were incubated under standard fusion conditions in the presence or absence of ATP. 1 mM DTT was added to oxidized vacuoles prior to the addition of ATP (oxidized/reduced). After 5 min of incubation at 27°C, vacuoles were solubilized in PS buffer either containing DTT for the reduced samples or without DTT for the oxidized samples and immuno-adsorbed to anti-Nyv1 protein A beads. Bound proteins were separated by SDS-PAGE, blotted, and probed with indicated antibodies. The DTT treatment of oxidized vacuoles in the detergent extract preserved the Nyv1/Vam7/Vti1 association in vacuoles that had undergone ATP-dependent priming. Thus, *cis*-SNARE complexes decay in the solublizate unless protected by DTT. This explains why their persistence after priming was not recognized in numerous previous studies [3]–[6]. Deliberate oxidation, by contrast, appears to destabilize *cis*-SNARE complexes already in the vacuolar membrane, and thereby prevents fusion.
(TIF)

Figure S10 Vam7-expression levels in spheroplast derived from wild type, *vps41Δ*, *ypt7T22N*, and *ccz1Δ* strains. 50 µg of protein from spheroblasts were loaded on a SDS-PAGE and separated proteins were blotted and probed for the indicated proteins.
(TIF)

Table S1 Yeast strains used in this study.
(PDF)

Text S1 Supplementary Materials and Methods.
(DOC)
