# Supplementary figures and images for: Sequential Analysis of Trans-SNARE Formation in Intracellular Membrane Fusion
Source: PLoS Biol. 2012 Jan 17;10(1):e1001243. doi: 10.1371/journal.pbio.1001243 (PMC3260307; doi:10.1371/journal.pbio.1001243)

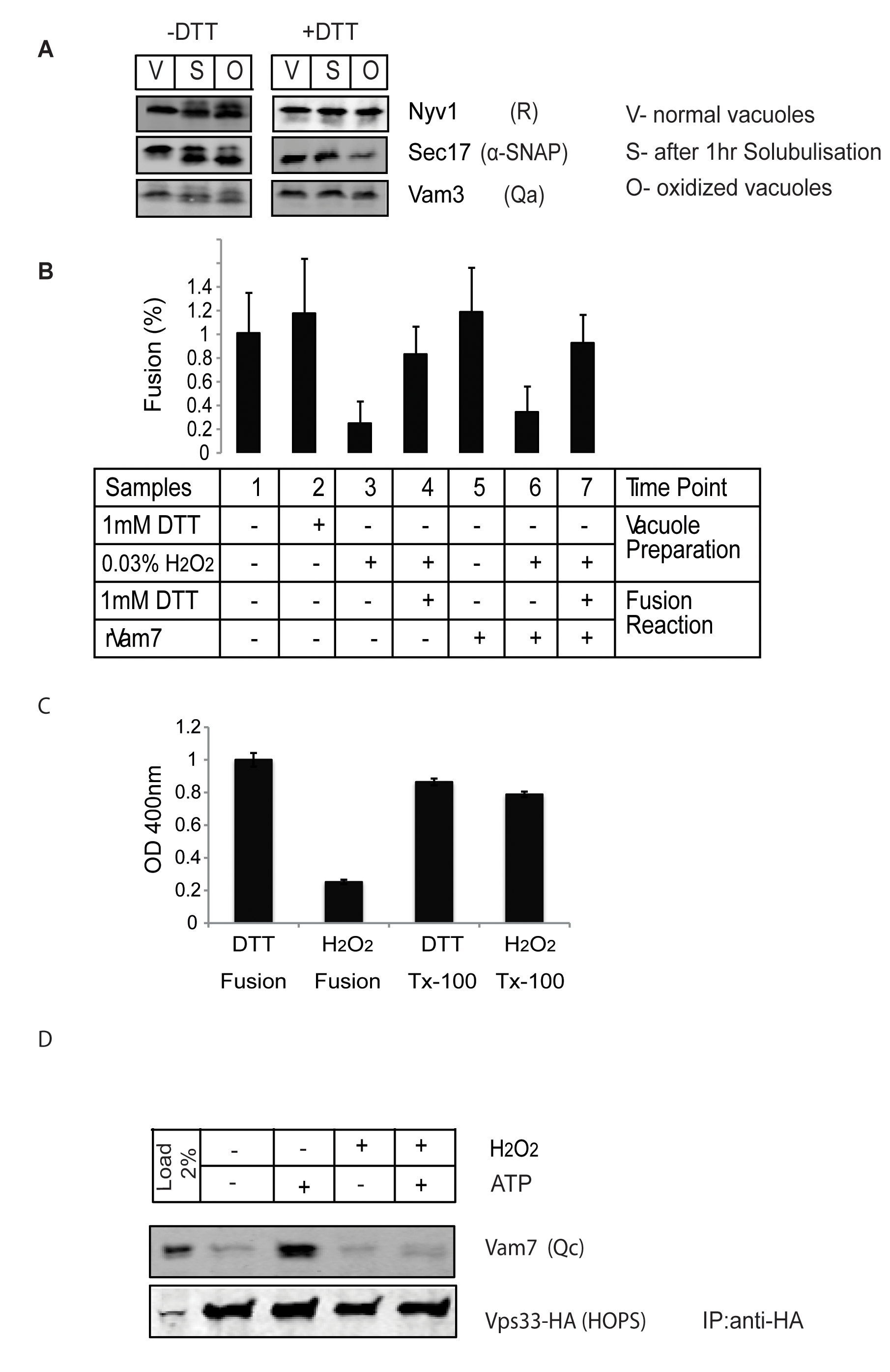

Supplement: Figure S1 — Effect of oxidation on mobility of SNAREs on SDS-PAGE, HOPS-SNARE binding, and fusion. (A) Oxidized or normal vacuoles were harvested from step gradients in the presence or absence of 0.03% (v/v) H2O2. An equal amount of vacuoles was directly mixed with SDS sample buffer with (right panel) or without (left panel) DTT. Another sample of equal amount of normal vacuoles was solubilized with Triton X-100 for 1 h at 4°C. Subsequently, a sample of the solubilizate was mixed with SDS buffer with or without DTT. These samples were separated by SDS-PAGE, blotted, and probed with the indicated antibodies. When run on a non-reducing SDS-gel, Nyv1, Vam3, and Sec17 displayed multiple bands (Figure S1A, left panel) depending on the time point at which vacuoles were picked. Vacuoles taken freshly from the ficoll gradient showed little or no oxidation, whereas vacuoles deliberately oxidized during centrifugation by adding H2O2, or samples taken from the solubilizate, contained significant amounts of oxidized proteins. In contrast to Vti1 and Vam7, the three aforementioned proteins comprise multiple cysteines and are therefore prone to oxidation. The multiple band patterns is not due to proteolysis since the presence of DTT in the SDS-sample buffer leads to the occurrence of only one single band for each displayed protein (Figure S1A, right panel). Due to this observation and the fact that the cytosol normally represents a reducing environment, we explored the influence of the redox conditions on SNARE behavior and fusion activity. (B) The cell-free fusion of yeast vacuoles is traced via maturation of the pro-alkaline phosphatase pro-Pho8p in one fusion partner by the maturase Pep4p contained in the other fusion partner. In order to create defined conditions before the start of fusion, we oxidized vacuoles by inclusion of 0.03% H2O2 (v/v) into the buffers for vacuole isolation, and we also prepared reduced vacuoles by including 1 mM DTT. The H2O2 concentration was chosen based on th [file pbio.1001243.s001.tif]

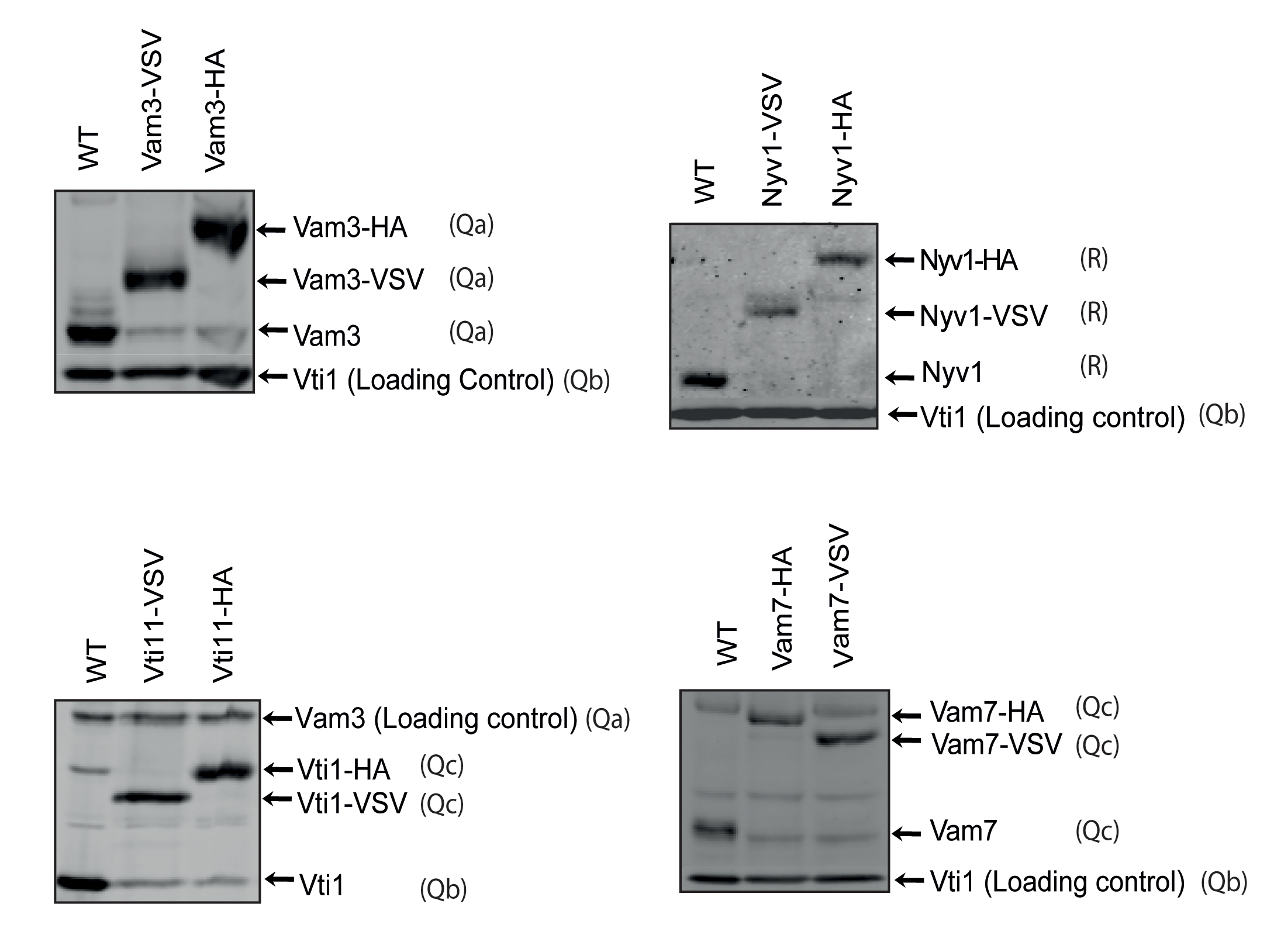

Supplement: Figure S2 — Expression rates of tagged SNAREs. For each tagged SNARE version, 30 µg of vacuoles were loaded on a SDS-PAGE followed by Western blotting with indicated antibodies against SNAREs. The added tags resulted in different running velocities of the SNARE proteins. (TIF) [file pbio.1001243.s002.tif]

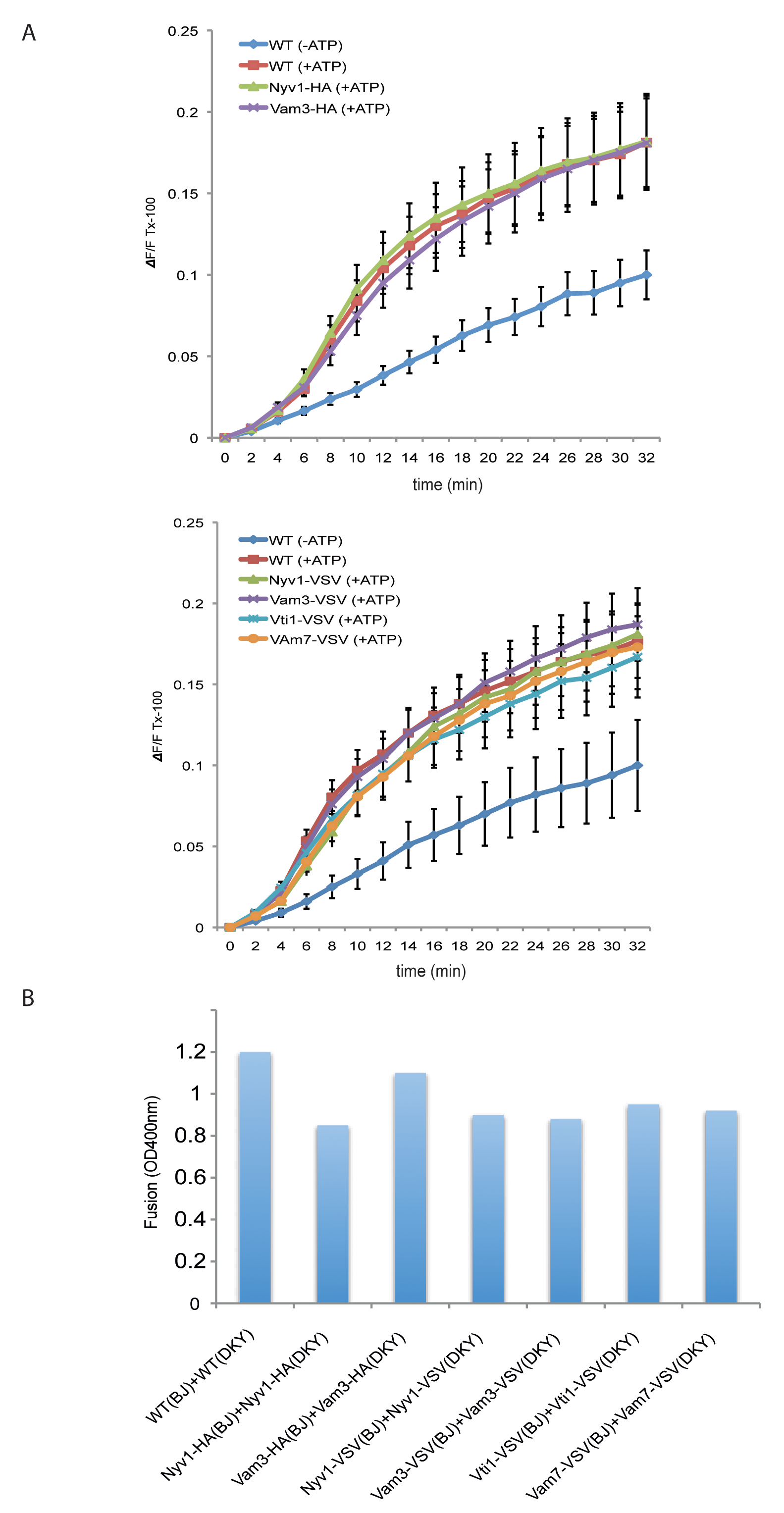

Supplement: Figure S3 — Fusion rates of vacuoles harboring tagged SNARE-versions. Fusion rates of vacuoles harboring tagged SNARE versions were measured by the standard hemifusion assay [2] and vacuole fusion assay. In both experiments, fusion rates of wildtype and tagged SNARE vacuoles were comparable. (TIF) [file pbio.1001243.s003.tif]

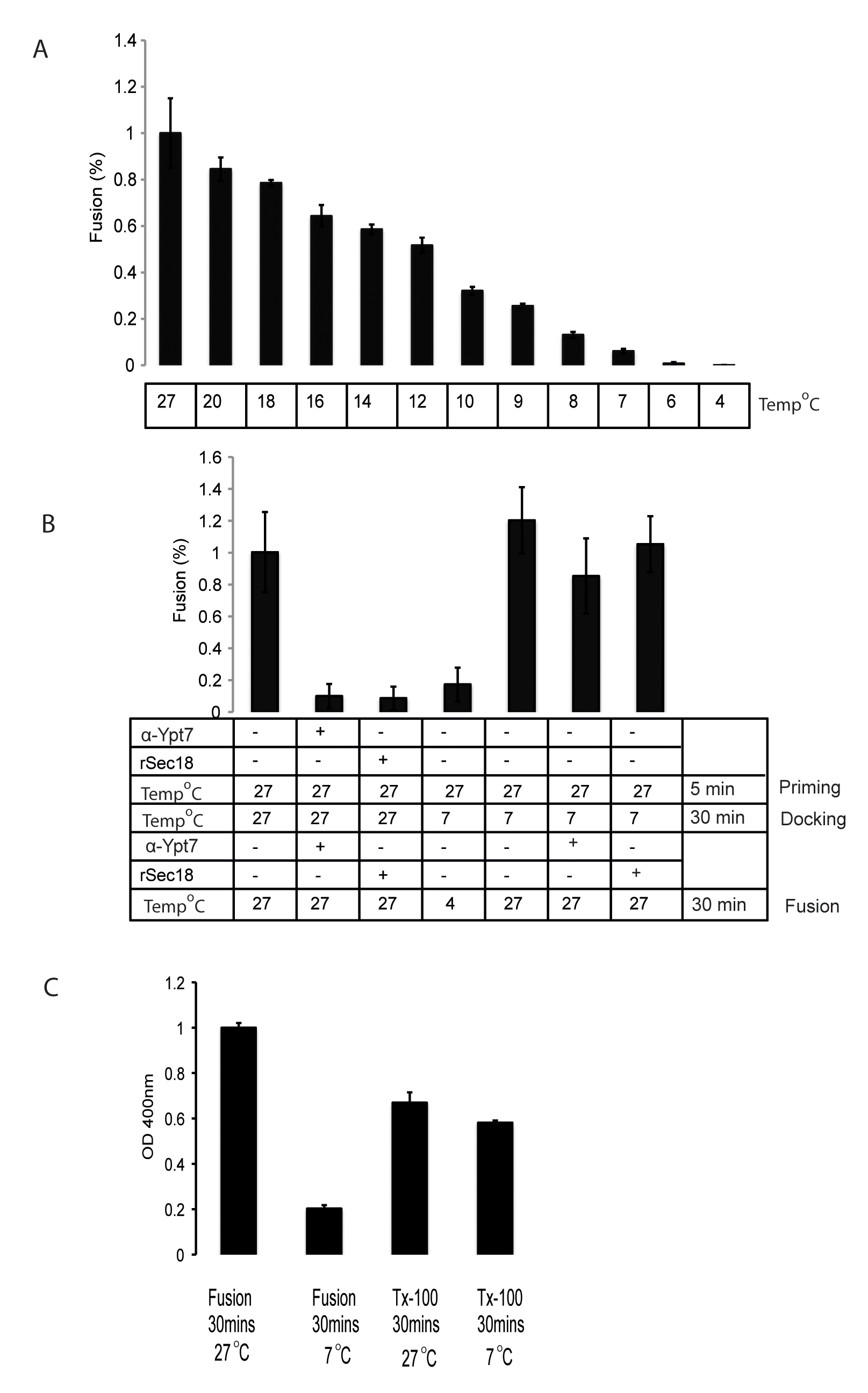

Supplement: Figure S4 — Temperature dependence of fusion stages and fusion rates. (A) Temperature titration of standard vacuolar fusion reactions. Content mixing almost stops completely at 7°C. All samples were primed for 5 min at 27°C prior to the incubation at lower temperatures. (B) Incubation of vacuoles at 7°C for 30 min allows completion of docking but prevents content mixing. Vacuoles were incubated with (Lane 2&3) or without inhibitor for 5 min at 27°C (priming). Then the incubation was continued at 27°C (Lanes 1–3) or at 7°C (Lanes 4–7) for 30 min in the presence or absence of inhibitors (docking). Thereafter, inhibitors were added to Lanes 6 and 7. Subsequently all samples, except those of the 7°C control (lane 4), were shifted to 27°C and further incubated for 30 min (Fusion). (C) Processing of ALP is not dramatically affected by lowering temperatures. Vacuoles were either incubated for 30 min under standard fusion conditions at 27°C or 7°C in order to assay fusion or for 1 h at 27°C or 7°C in the presence of 0.2% Triton X-100 in order to assay reporter maturation. (TIF) [file pbio.1001243.s004.tif]

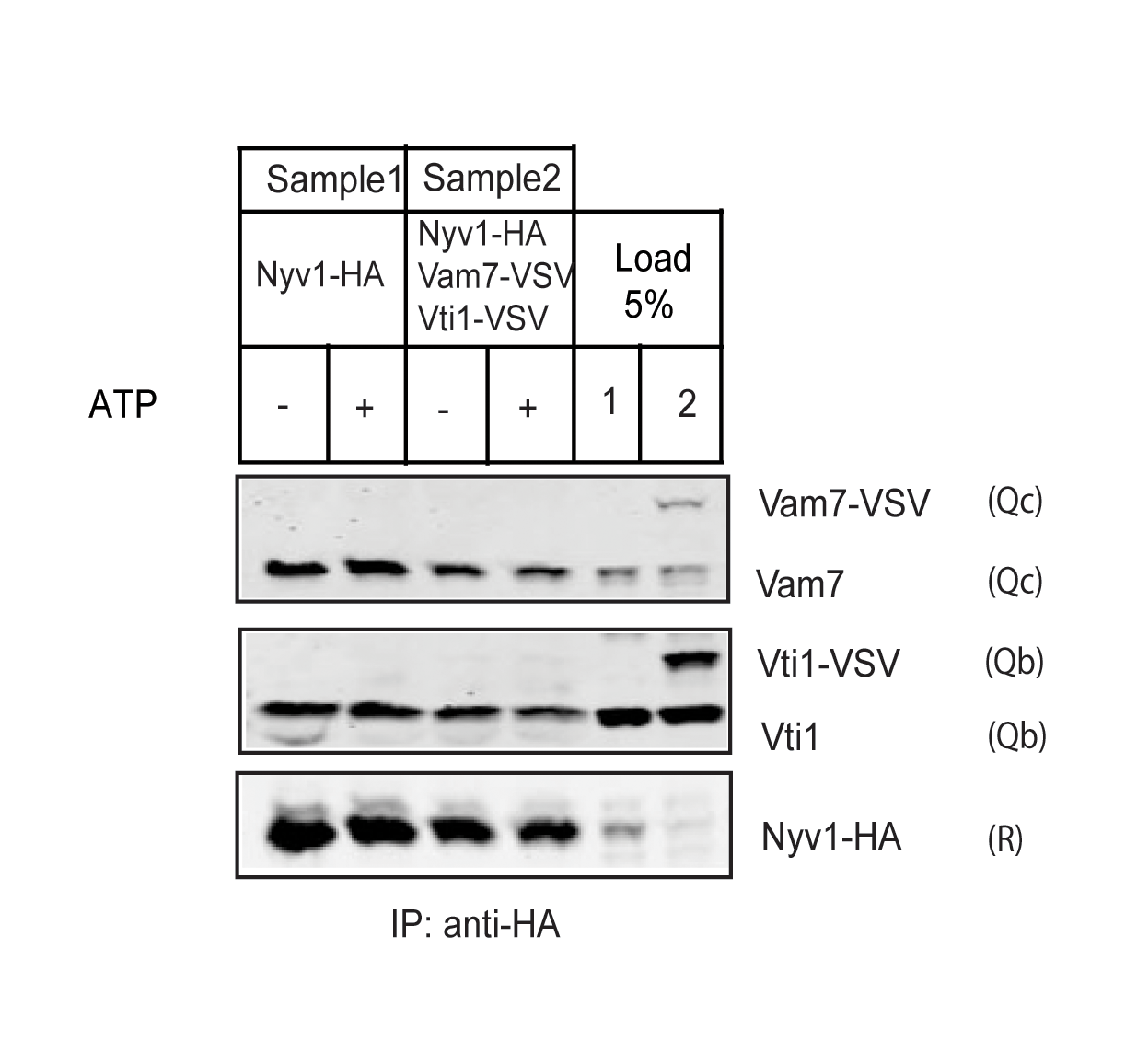

Supplement: Figure S5 — SNAREs do not reassemble randomly into new complexes in the detergent extract. Vacuoles from strains harboring Nyv1-HA, Vti1-VSV, and Vam7-VSV were purified and primed under standard conditions. After separate solubilization, Nyv1-HA was precipitated either from samples containing only Nyv1-HA (100%) or from a detergent extract containing a mixture of Nyv1-HA, Vam7-VSV, and Vti1-VSV (50%, 25%, 25%). We did not observe any intermixing of Vam7-VSV or VTI1-VSV with Nyv1-HA in the detergent extract, indicating the stability of the vacuolar QbcR-complex. (TIF) [file pbio.1001243.s005.tif]

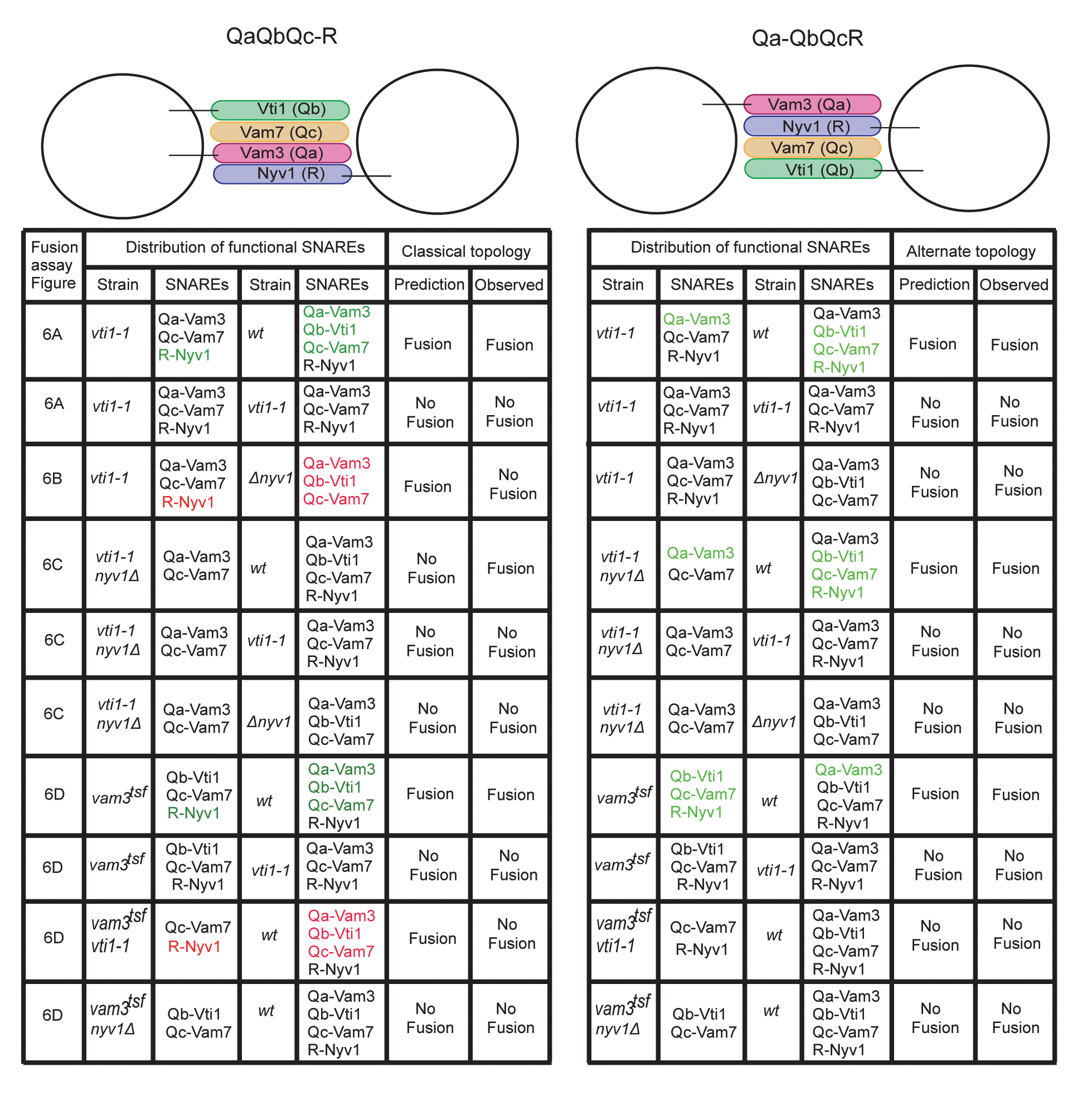

Supplement: Figure S6 — Combinations of v-SNARE deletions and conditional t-SNARE alleles to distinguish the Qa-QbcR and Qabc-R topologies. Vacuoles were isolated from strains carrying the indicated combinations of deletions or temperature-sensitive alleles. The table depicts a comparison of observed fusion effects with the predictions by the Qa-QbcR and Qabc-R models. Green colored SNARE combinations show fusion; red colored SNARE combinations show no fusion. (TIF) [file pbio.1001243.s006.tif]

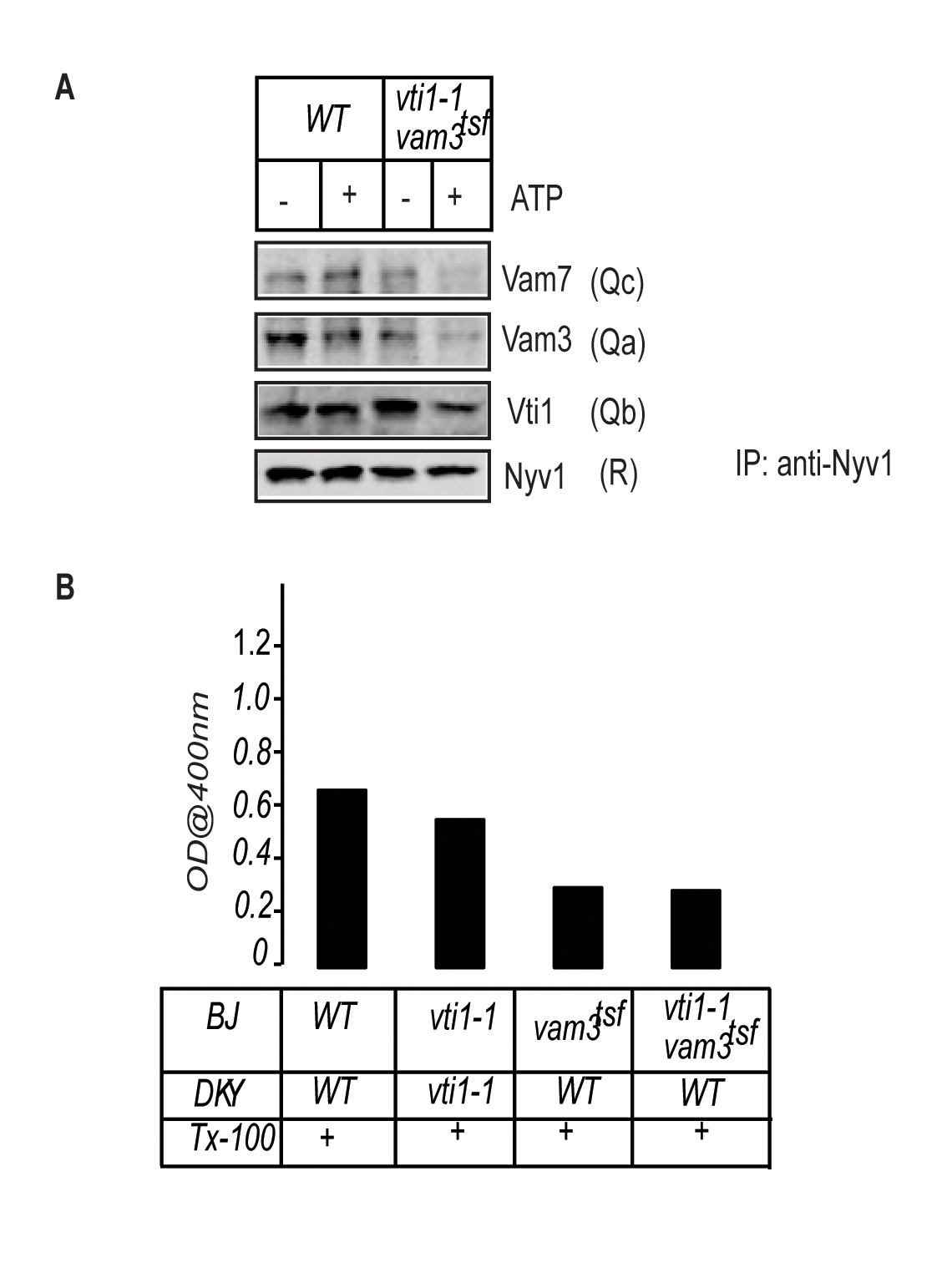

Supplement: Figure S7 — The vti1-1 vam3tsf double mutant primes normally, but displays an unstable QbcR-complex. (A) Vacuoles from wildtype and vti1-1 vam3tsf mutant cells were purified under non-permissive conditions and QbcR-complex stability was assayed as described above. Wildtype vacuoles displayed the expected stable QbcR-complex, whereas vacuoles derived from the double mutant showed loss of most of the Vam7 and Vti1 from Nyv1, indicating that priming works but post-priming QbcR-complex stability is lost. (B) Reporter control for different SNAREts mutants: BJ and DKY vacuoles were purified from the indicated strains and incubated for 60 min at 27°C in the presence of 0.2% TritonX-100. All BJ mutants harboring the vam3tsf mutation showed only 50% ALP loading compared to wildtype vacuoles, which was taken into account by a longer incubation time in developing buffer. (TIF) [file pbio.1001243.s007.tif]

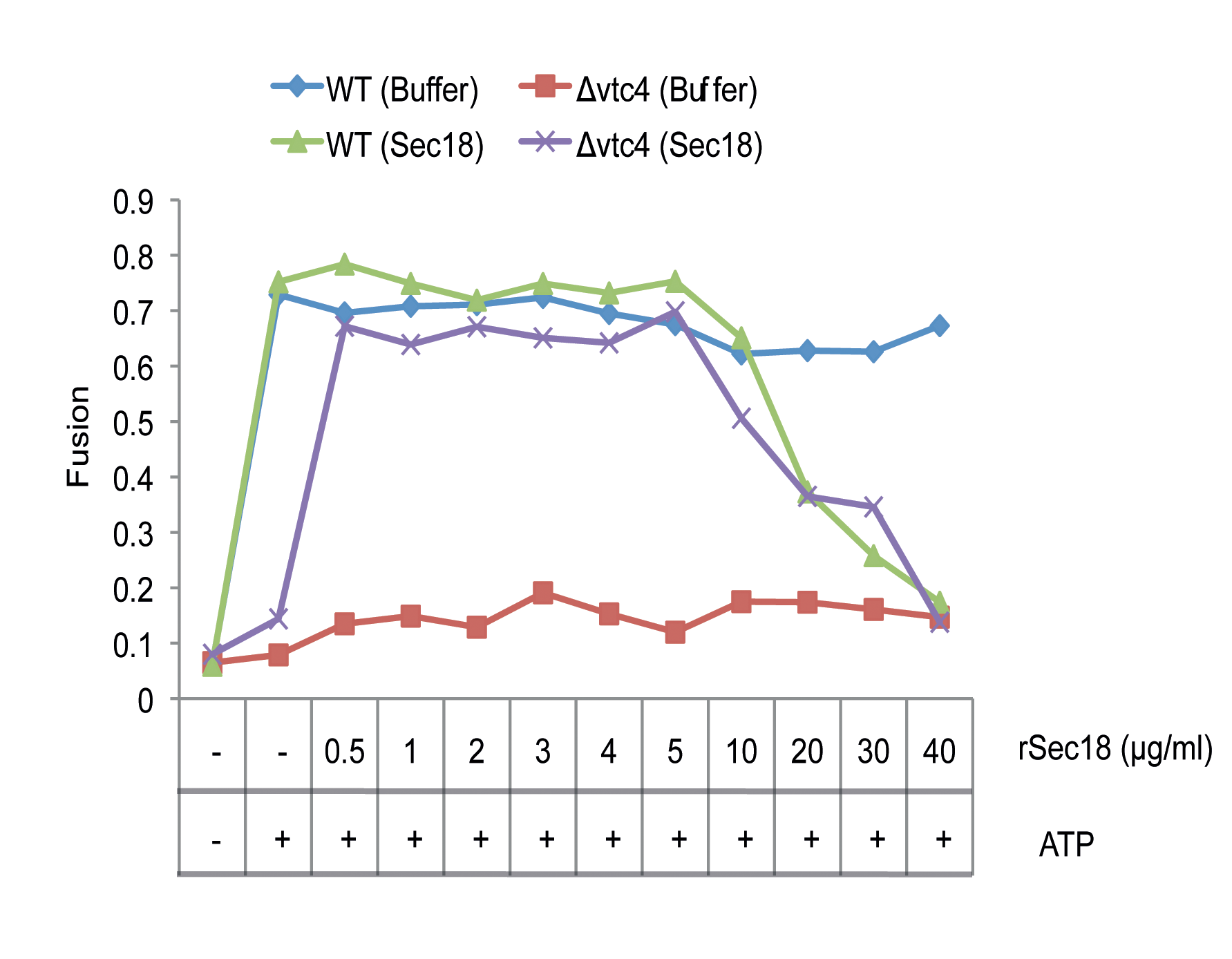

Supplement: Figure S8 — rSec18 influence on vacuolar fusion at different concentrations. Vacuoles from wildtype cells (BJ&DKY) or vtc4Δ (BJ&DKY) were incubated under fusion conditions in the presence or absence of ATP and rSec18. Recombinant Sec18 was added at increasing concentration ranging from 1 µg/ml up to 40 µg/ml. As a buffer control, rSec18 samples were heat inactivated, and the remaining supernatant added to the fusion reaction with the same volume as active rSec18. After 60 min of incubation at 27°C fusion, activity was assayed as described in “Materials and Methods.” (TIF) [file pbio.1001243.s008.tif]

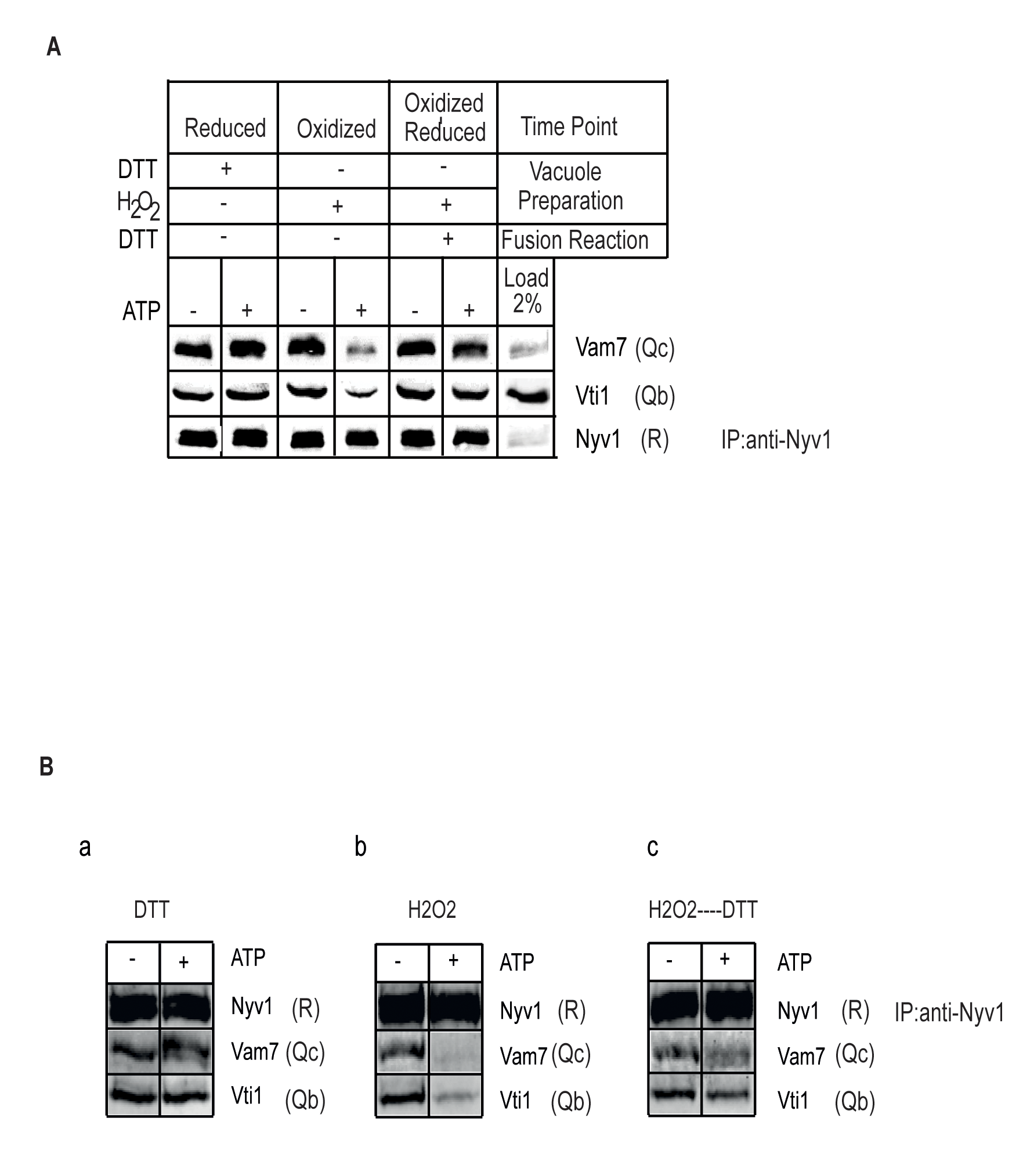

Supplement: Figure S9 — Influence of oxidation on the persisting QbcR-complex. (A) Nyv1 was precipitated from oxidized and reduced vacuoles or from vacuoles that were oxidized during the isolation procedure but were complemented with DTT in the fusion reaction. In the absence of ATP, Vti1 and Vam7 co-fractionated with Nyv1 under all conditions. ATP addition efficiently separated Vam7 and Vti1 from Nyv1 for the oxidized sample, consistent with published observations. However, the association of Vam7 and Vti1 with Nyv1 persisted even after ATP incubation for the reduced sample. Oxidized vacuoles that had been primed under reducing conditions behaved in a similar manner as those that were kept under reducing conditions throughout. This suggests that the destabilization of cis-SNARE complexes by oxidation of vacuoles is reversible. (B) Oxidation of proteins mainly occurs in the detergent extract. Vacuoles prepared in the absence of DTT fuse almost as efficiently as vacuoles prepared in its presence (Figure S1B). Therefore, we asked whether, in the absence of deliberate oxidation of the vacuoles by H2O2, the cis-SNARE complexes might remain stable enough during priming and docking, but decay subsequently in the solubilizate. Oxidized and reduced vacuoles were harvested from gradients containing H2O2 or DTT. Vacuoles were incubated under standard fusion conditions in the presence or absence of ATP. 1 mM DTT was added to oxidized vacuoles prior to the addition of ATP (oxidized/reduced). After 5 min of incubation at 27°C, vacuoles were solubilized in PS buffer either containing DTT for the reduced samples or without DTT for the oxidized samples and immuno-adsorbed to anti-Nyv1 protein A beads. Bound proteins were separated by SDS-PAGE, blotted, and probed with indicated antibodies. The DTT treatment of oxidized vacuoles in the detergent extract preserved the Nyv1/Vam7/Vti1 association in vacuoles that had undergone ATP-dependent priming. Thus, cis-SNARE complexes decay in the solublizate unless pr [file pbio.1001243.s009.tif]

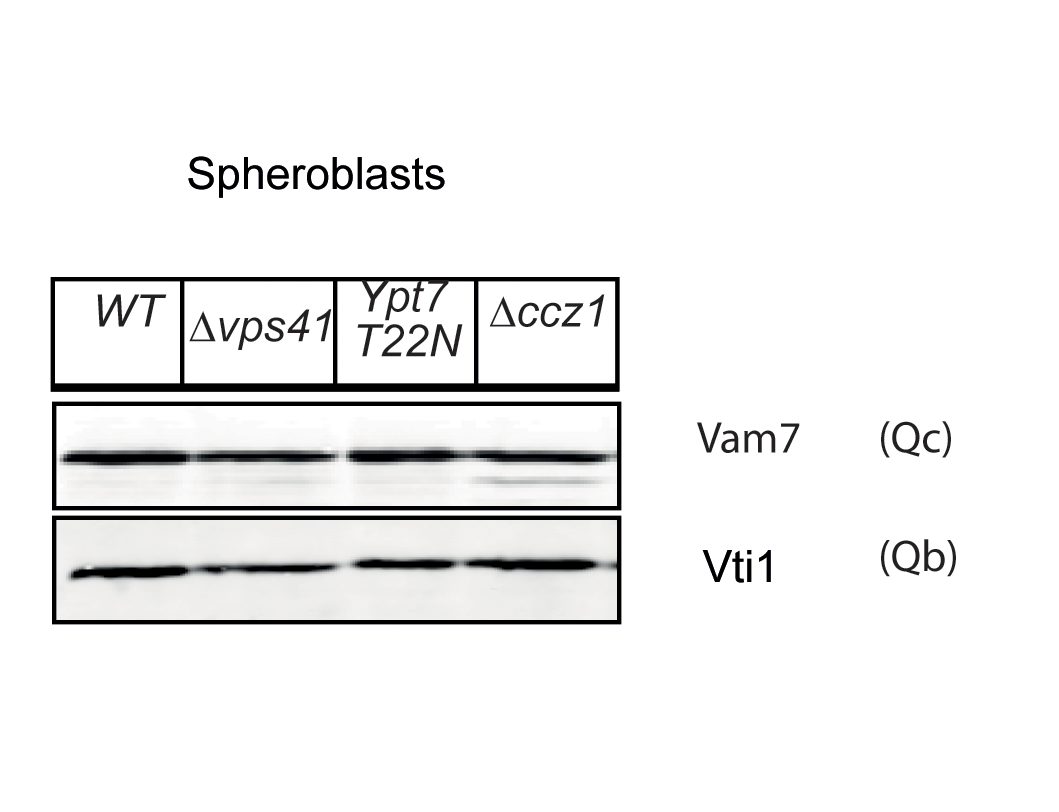

Supplement: Figure S10 — Vam7-expression levels in spheroplast derived from wild type, vps41Δ, ypt7T22N, and ccz1Δ strains. 50 µg of protein from spheroblasts were loaded on a SDS-PAGE and separated proteins were blotted and probed for the indicated proteins. (TIF) [file pbio.1001243.s010.tif]
