# Supplementary material for: Sequential Analysis of Trans-SNARE Formation in Intracellular Membrane Fusion
Source: PLoS Biol. 2012 Jan 17;10(1):e1001243. doi: 10.1371/journal.pbio.1001243 (PMC3260307; doi:10.1371/journal.pbio.1001243)
Supplement: Table S1 — Yeast strains used in this study. (PDF) [file pbio.1001243.s011.pdf]

Table S1.Yeast strains used in this study

Strain	Genotype	Reference
BJ3505	*MATa pep4::HIS3 prb1-Δ1.6R lys2-208 trp1-Δ101 ura3-52 gal2 can*	[12]
DKY6281	*MATα leu2-3 leu2-112 ura3-52 his3-Δ200 trpl-Δ901 lys2-801 suc2-Δ9 pho8::TRP1*	[13]
BJ Nyv1-HA	BJ 3505; nyv1-His6(HA)3 ::Nat	This study
BJ Vam3-HA	BJ 3505; vam3-His6(HA)3 ::Nat	This study
DKY Nyv1-HA	DKY 6281; nyv1-His6(HA)3 ::Nat	This study
DKY Vam3-HA	DKY 6281; Vam3-His6(HA)3 ::Nat	This study
BJ nyv1Δ	BJ3505; nyv1::TRP1	[14]
DKY nyv1Δ	DKY6281; nyv1::URA	[14]
BJ nyv1Δ vti1-1	BJ *vti1-1*; nyv1::TRP1	This study
BJ Vam3^{tsf} vti1-1	BJ *vti1-1*; vam3::URA; Vam3^{tsf} ::Nat	This study
BJ Nyv1-VSV	BJ 3505; nyv1-His6-VSV ::kanmx	This study
BJ Vam3-VSV	BJ 3505; vam3-His6-VSV ::kanmx	This study
BJ Vam7-VSV	BJ 3505; vam7-His6-VSV ::kanmx	This study
BJ Vti1-VSV	BJ 3505; vti1-His6-VSV ::kanmx	This study
BJ ccz1Δ	BJ 3505; ccz1::kanMX	[15]
BJ ypt7T22N	BJ 3505; ypt7::Nat	This study
BJ vps41Δ	BJ 3505; vps41::Nat	This study
BJ-vam7	BJ 3505; pRS416-vam7	This study
BJ ccz1Δ-vam7	BJ *ccz1Δ*; pRS416-vam7	This study
BJ ypt7T22N-vam7	BJ ypt7;pRS416-vam7	This study
BJ vps41Δ-vam7	BJ *vps41Δ*; pRS416-vam7	This study
DKY Nyv1-VSV	DKY 6281; nyv1-His6-VSV ::kanmx	This study
DKY Vam3-VSV	DKY 6281; vam3-His6-VSV ::kanmx	This study
DKY Vam7-VSV	DKY 6281; vam7-His6-VSV ::kanmx	This study
DKY Vti1-VSV	DKY 6281; vti1-His6-VSV ::kanmx	This study
DKY ccz1Δ	DKY 6281; ccz1::kanMX	[15]
DKY ypt7T22N	DKY 6281; ypt7::Nat	This study
DKY vps41Δ	DKY 6281; vps41::Nat	This study
DKY vam7	DKY 6281; pRS416-vam7	This study
DKY ccz1Δ-vam7	DKY *ccz1Δ*; pRS416-vam7	This study
DKY ypt7T22N-vam7	DKY *ypt7T22N*;pRS416-vam7	This study
DKY vps41Δ-vam7	DKY *vps41Δ*; pRS416-vam7	This study
