# Supplementary material for: Sequential Analysis of Trans-SNARE Formation in Intracellular Membrane Fusion
Source: PLoS Biol. 2012 Jan 17;10(1):e1001243. doi: 10.1371/journal.pbio.1001243 (PMC3260307; doi:10.1371/journal.pbio.1001243)
Supplement: Text S1 — Supplementary Materials and Methods. (DOC) [file pbio.1001243.s012.doc]

**SUPPORTING INFORMATION**

**Sequential analysis of trans-SNARE formation in intracellular membrane fusion**

Kannan Alpadi^1^, Veronique Comte^2^, Monique Reinhardt^2^, Andrea Schmidt^2^, Sarita Namjoshi^1^, Aditya Kulkarni^1^, Andreas Mayer^2^ & Christopher Peters^1^

^1^Verna and Marrs McLean Department of Biochemistry and Molecular Biology, Baylor College of Medicine, Houston, TX, 77030 and ^2^Département de Biochimie, Université de Lausanne, Chemin des Boveresses 155, 1066 Epalinges, Switzerland

Phone: 001-7137981876

Fax: 001-7137969438

E-mail: cpeters@bcm.edu

**Supplementary materials and methods**

*Strains*

BJ3505 (*MATa pep4::HIS3 prb1-∆1.6R his3 lys2-208 trp1-∆101 ura3-52 gal2 can*) and DKY6281 (*MATa leu2-3, -112 ura3-52 his3-∆200 trp1-∆901 lys2-801 suc2-∆9 pho8::TRP1*) were the standard fusion strains. C-terminal peptide tags were introduced by one-step gene replacement using standard PCR-mediated techniques and a KAN-MX or NAT marker [7]. BJ3505 *vti1-1* and DKY6181 *vti1-1* are described [8]. *Vam3^tsf^* was introduced into these strains by deleting endogenous Vam3 and introducing a CEN-based plasmid [9] expressing *vam3^tsf^* or wild type Vam3 from its native promoter. For generating the *nyv1Δ vam3^tsf^* double mutant, *vam3^tsf^* was introduced in *nyv1Δ vam3Δ* mutant cells.

*Reagents*

GDI was expressed and purified as described in Garrett et al., 1994 [10]. In the final step, fractions were pooled and concentrated in PS-buffer containing 125mM KCl. Polyclonal antisera were raised in rabbits using purified recombinant proteins from Escherichia coli. Monoclonal antibodies were purchased for HA from Covance (HA-acites). Sec18 was purified as described in [11].

**References**

1. Thorngren N, Collins KM, Fratti RA, Wickner W, Merz AJ (2004) A soluble SNARE drives rapid docking, bypassing ATP and Sec17/18p for vacuole fusion. EMBO J 23: 2765-2776.

2. Pieren M, Schmidt A, Mayer A (2010) The SM protein Vps33 and the t-SNARE H(abc) domain promote fusion pore opening. Nat Struct Mol Biol 17: 710-717.

3. Ungermann C, Nichols BJ, Pelham HR, Wickner W (1998) A vacuolar v-t-SNARE complex, the predominant form in vivo and on isolated vacuoles, is disassembled and activated for docking and fusion. J Cell Biol 140: 61-69.

4. Muller O, Bayer MJ, Peters C, Andersen JS, Mann M, et al. (2002) The Vtc proteins in vacuole fusion: coupling NSF activity to V(0) trans-complex formation. EMBO J 21: 259-269.

5. Reese C, Heise F, Mayer A (2005) Trans-SNARE pairing can precede a hemifusion intermediate in intracellular membrane fusion. Nature *436*: 410-414.

6. Collins, K. M., Thorngren, N. L., Fratti, R. A., and Wickner, W. T. (2005). Sec17p and HOPS, in distinct SNARE complexes, mediate SNARE complex disruption or assembly for fusion. EMBO J *24*: 1775-1786.

7. Janke C, Magiera MM, Rathfelder N, Taxis C, Reber S, et al. (2004) A versatile toolbox for PCR-based tagging of yeast genes: new fluorescent proteins, more markers and promoter substitution cassettes. Yeast *21*: 947-962.

8. Ungermann C, von Mollard GF, Jensen ON, Margolis N, Stevens TH, et al. (1999) Three v-SNAREs and two t-SNAREs, present in a pentameric cis-SNARE complex on isolated vacuoles, are essential for homotypic fusion. J Cell Biol 145: 1435-1442.

9. Darsow T, Rieder SE, Emr SD (1997) A multispecificity syntaxin homologue, Vam3p, essential for autophagic and biosynthetic protein transport to the vacuole. J Cell Biol 138: 517-529.

10. Garrett MD, Zahner JE, Cheney, CM, Novick PJ (1994) GDI encodes a GDP dissociation inhibitor that plays an essential role in the yeast secretory pathway. EMBO J 13:1718-28.

# 11. Xu Z, Sato K, Wickner W (1998) LMA1 binds to vacuoles at Sec18p (NSF), transfers upon ATP hydrolysis to a t-SNARE (Vam3p) complex, and is released during fusion. Cell 93:1125-34

12.Jones EW, Zubenko GS, Parker RR (1982) PEP4 gene function is required for expression of several vacuolar hydrolases in Saccharomyces cerevisiae. Genetics 102: 665-77.

13. Haas A, Conradt B. Wickner W(1994) G-protein ligands inhibit in vitro

reactions of vacuole inheritance. J Cell Biol 126: 87-97.

14. Nichols BJ, Ungermann C, Pelham HRB, Wickner W et al. (1997) Homotypic vacuolar fusion mediated by v- and t-SNAREs. Nature 387: 199–202.

15. Baars TL, petri S, Peters C, Mayer A (2007) Role of the V-ATPase in regulation of the vacuolar fission-fusion equilibrium. Mol Biol Cell 10: 3873-82
